# Emergent behaviour and neural dynamics in artificial agents tracking odour plumes

**DOI:** 10.1038/s42256-022-00599-w

**Published:** 2023-01-25

**Authors:** Satpreet H. Singh, Floris van Breugel, Rajesh P. N. Rao, Bingni W. Brunton

**Affiliations:** 1 University of Washington, Seattle, WA, USA.; 2 University of Nevada, Reno, NV, USA.

## Abstract

Tracking an odour plume to locate its source under variable wind and plume statistics is a complex task. Flying insects routinely accomplish such tracking, often over long distances, in pursuit of food or mates. Several aspects of this remarkable behaviour and its underlying neural circuitry have been studied experimentally. Here we take a complementary in silico approach to develop an integrated understanding of their behaviour and neural computations. Specifically, we train artificial recurrent neural network agents using deep reinforcement learning to locate the source of simulated odour plumes that mimic features of plumes in a turbulent flow. Interestingly, the agents’ emergent behaviours resemble those of flying insects, and the recurrent neural networks learn to compute task-relevant variables with distinct dynamic structures in population activity. Our analyses put forward a testable behavioural hypothesis for tracking plumes in changing wind direction, and we provide key intuitions for memory requirements and neural dynamics in odour plume tracking.

## Reporting summary

Further information on research design is available in the Nature Portfolio Reporting Summary linked to this article.

Locating the source of an odour in a windy environment is a challenging control problem, where an agent must act to correct course in the face of intermittent odour signals, changing wind direction and variability in odour plume shape^1,2^. Moreover, an agent tracking an intermittent plume needs memory, where current and past egocentric odour, visual and wind sensory signals must be integrated to determine the next action. For flying insects, localizing the source of odour plumes emanating from potential food sources or mates is critical for survival and reproduction. Therefore, many aspects of their plume tracking abilities have been experimentally studied in great detail^3–5^. However, most such studies are limited to one or two levels of analysis, such as behaviour^6^, computation^7,8^ or neural implementation^9^.

Despite the wide adoption of wind tunnel experiments to study odour plume tracking^10^, generating controlled dynamic odour plumes in turbulent flow and recording flight trajectories at high resolution is expensive and laborious. Exciting alternative approaches have been developed using virtual reality^11^ and kilometre-scale outdoor dispersal experiments^12^. While behavioural experiments are now tractable, collecting substantial neural data during free flight in small insects remains technologically infeasible, and larger insects require larger wind tunnels. Here we are motivated to take a complementary in silico approach using artificial recurrent neural network (RNN) agents trained to track simulated odour plumes that mimic features of plumes evolving in turbulent flow, with the goal of developing an integrated understanding of the behavioural strategies and the associated neural computations that support plume tracking.

**Fig. 1 |:**
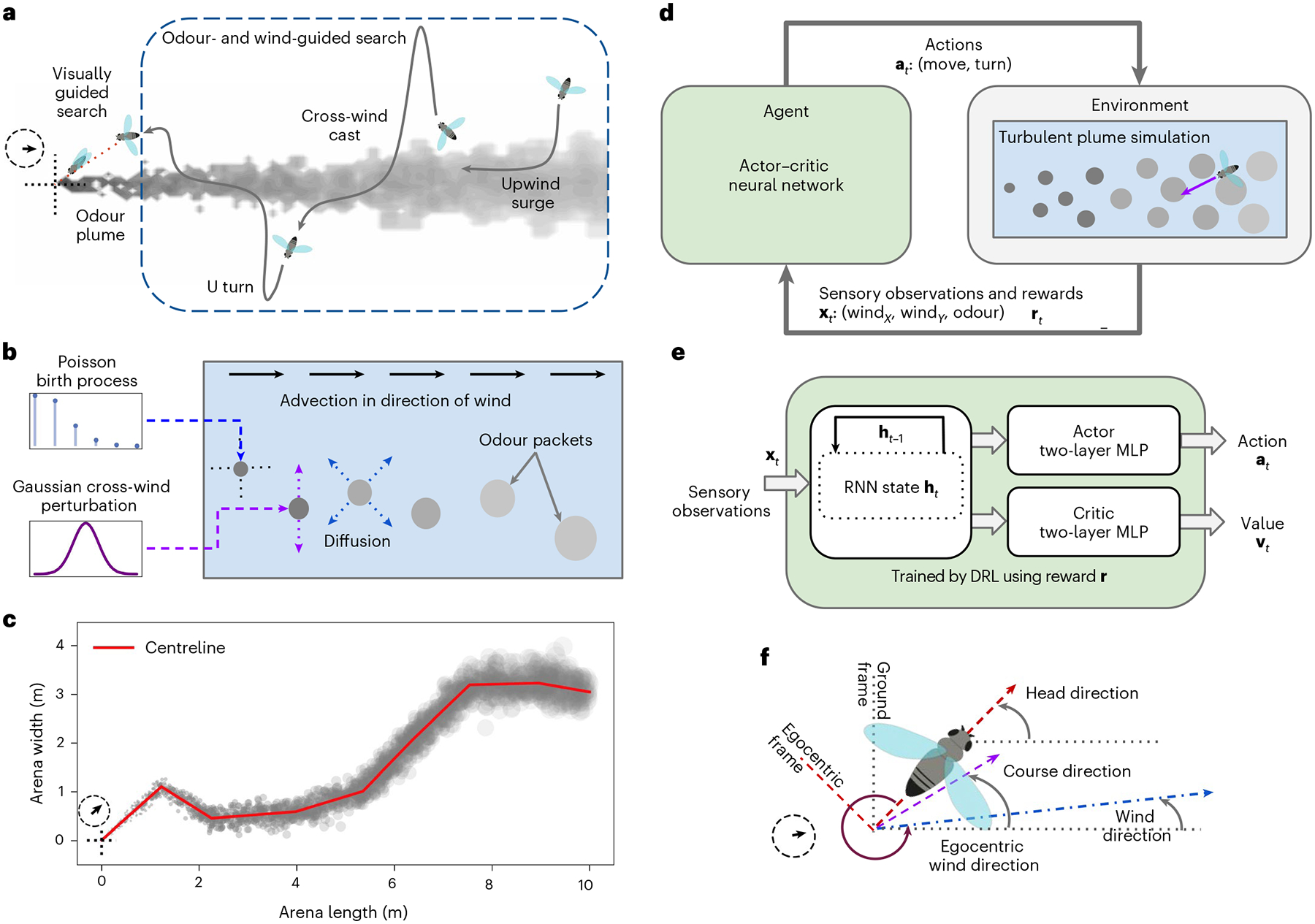
Training artificial agents to track dynamic odour plumes with DRL. **a**, A schematic of a flying insect performing a plume tracking task, showing upwind surge, cross-wind cast and U-turn behaviours. In this work, we model the spatial scale (dashed rectangle) where the insect can use only olfactory and mechanosensory wind sensing cues for plume tracking. **b**, The plume simulator models stochastic emission of odour packets from a source carried by wind. Odour packets are subject to advection by wind, random cross-wind perturbation and radial diffusion. **c**, An example of a plume simulation where the wind direction changed several times. The centreline of the plume is in red. **d**, A schematic of how the artificial agent interacts with the environment at each time step. The plume simulator model of the environment determines the sensory information **x** (egocentric wind-direction vector and local odour concentration) available to the agent and the rewards used in training. The agent navigates within the environment with actions **a** (turn direction and magnitude of movement). **e**, Agents are modelled as neural networks and trained by DRL. An RNN generates an internal state representation **h** from sensory observations, followed by parallel actor and critic heads that implement the agent’s control policy and predict the state values, respectively. The actor and critic heads are two-layer, feedforward MLP networks. **f**, A schematic to illustrate an agent’s head direction and course direction and the wind direction, all measured with respect to the ground and anticlockwise from the *x* axis. Course direction is the direction in which the agent actually moves, accounting for the effect of the wind on the agent’s intended direction of movement (head direction). Egocentric wind direction is the direction of the wind as sensed by the agent. Panels **a**,**f** adapted with permission from ref.^98^ under a Creative Commons licence CC BY 4.0. Panel **a** inspired by a figure in Baker et al.^3.^

In recent years, artificial neural networks (ANNs) have gained increasing popularity for modelling and understanding aspects of neural function and animal behaviour including vision^13^, movement^14^ and navigation^15,16^. Whereas many ANNs have been trained using supervised approaches that rely on labelled training data, an alternative emerging algorithmic toolkit known as deep reinforcement learning (DRL) has made it computationally tractable to train ANN agents (Fig. 1d). In particular, an ANN agent receives sensory observations and task-aligned rewards based on its actions at each step and tries to learn a strategy for its next actions to maximize total expected reward^17^. Such learning- and optimization-based models are normative in the sense that they can prescribe how a neural system should behave, rather than describing how it has been observed to behave. As neuroscience moves towards studying increasingly naturalistic behaviours^18,19^, such normative approaches are gaining traction as tools to gain insight, rapidly explore hypotheses and generate ideas for theoretical development^20–24^.

Flying insects search for sources of odour using several strategies, depending on the spatial scale being considered and odour source visibility^3^ (Fig. 1a). Close to the odour source, insects can fly to the source guided by vision. At longer ranges (from a few metres up to about 100 m; ref.^25^) or when the odour source is not yet visible, their search must be guided by olfaction to detect odours and mechanosensation to estimate wind velocity. At this larger scale, there are a few stereotyped behavioural sequences known to be important for plume tracking^5^: upwind surges when the insect can sense the odour, and cross-wind casts and U turns to locate the plume body when the insect loses the odour scent (Fig. 1a). Here we focus on this larger-scale odour- and wind-guided regime, where agents have access to only mechanosensory and olfactory cues.

In this Article, we describe behaviours that emerge in RNN agents trained to track odours in a flexible plume simulation and analyse the neural dynamics that underlie these behaviours. At a behavioural level, we find that the agents’ actions can be summarized by modules that closely resemble those observed in flying insects. While odour plumes that do not change in direction can be tracked using a few steps of history, longer timescales of memory are essential for plumes that are non-stationary and change direction unpredictably. Interestingly, the learned tracking behaviour of RNN agents in non-stationary plumes suggests a testable experimental hypothesis: that tracking is accomplished through local plume shape rather than wind direction. The RNNs learn to represent variables known to be important to flying insect navigation, such as head direction and time between odour encounters. Further, the low-dimensional neural activity associated with the emergent behaviour modules represents behaviourally relevant variables and is structured into two distinct regimes.

## Related work

In the field of neural computation, an emerging body of work has used DRL to train ANNs that solve tasks closely inspired by tasks from neuroscience. For instance, agents have been trained to study learning and dynamics in the motor cortex^26,27^, time encoding in the hippocampus^28^, reward-based learning and meta-learning in the prefrontal cortex^29–31^ and task-associated representations across multiple brain areas^32^. There have been several recent perspectives articulating the relevance of this emerging algorithmic toolkit to neuroscience^33,34^ and ethology^35^.

Our work is most directly related to three recent research efforts. Merel et al.^22^ developed a virtual-reality model of a rodent embodied in a skeleton body and endowed with a deep ANN ‘brain’. They trained this model using DRL to solve four tasks and then analysed the virtual rodent’s emergent behaviour and neural activity, finding similarities at an abstract level between their agent and observations from rodent studies. Reddy et al.^36^ studied the trail tracking strategies of terrestrial animals with one (for example one antenna) or two (for example two nostrils) odour sensors. They found that RL agents trained on simulated trails recapitulate the stereotypical zig-zagging tracking behaviour seen in such animals. Using a static trail model and an explicit (not neural) probabilistic model for sensory integration, they studied the effect of varying agent and task parameters on the emergent stereotypical zig-zagging behaviour. Rapp and Nawrot^37^ used a biologically detailed spiking neural circuit model of a fly mushroom body to study sensory processing, learning and motor control in flying insects when foraging within turbulent odour plumes.

We build on the approach of these recent papers that study artificial agents solving neural-inspired tasks, and our work is also distinct in several key ways. First, we simulate a more computationally challenging task than the static trail tracking task of Reddy et al.^36^, because our odour environment is configurable, dynamic and stochastic. In contrast, Rapp and Nawrot^37^ use a similar plume environment with only constant-wind-direction plumes, but with the added complexity of a secondary distractor odour that their agent must learn to avoid. Second, we have made several simplifications and abstractions that make analysis more tractable, so that we may focus on the general principles behind plume tracking. Specifically, we omit biomechanical details, impose no biologically inspired connectivity constraints and do not use spiking neurons. Instead, our networks are ‘vanilla’ RNNs (rather than the gated RNNs used by Merel et al.^22^ or the spiking neurons of Rapp and Nawrot^37^), which facilitates analyses from the dynamical systems perspective^38–42^. We analyse emergent behaviours and neural dynamics at the network level, which provides us with an abstract understanding of task-relevant neural computations that is robust to small changes in network architecture and training hyperparameters^39,41,42^. Finally but importantly, since we do not model vision or joint-level motor control as do Merel et al.^22^, our neural networks are simpler and can be trained on a computational budget accessible to an academic laboratory.

## Results

Our in silico agents learn strategies to successfully localize plume sources in non-stationary environments. In this section, we briefly summarize our approach and characterize agent performance, then highlight their emergent behavioural and neural features. In addition to comparing artificial agents with biology, we discover behavioural strategies that motivate future experiments and gain intuition about the neural computations underlying these emergent behaviours.

### Training artificial agents to track odour plumes

We use a particle-based two-dimensional plume model^43^, which is computationally tractable and can provide exemplars that are known to approximate features of real-world odour plumes such as intermittency, rapid fluctuations in instantaneous concentration, and Gaussian time-averaged cross-section concentration (Fig. 1b). Agents are actor–critic neural networks^44^ that receive continuous-valued sensory observations as inputs (that is, egocentric instantaneous wind velocity and local odour concentration) and produce continuous-valued move and turn actions (Fig. 1e). Parameters of the environment simulation and agent actions are roughly matched to the capability of flies. Training is done using the proximal policy optimization (PPO)^45^ algorithm, with agents initialized at random locations within or slightly outside plumes that switch directions multiple times during the course of the episode.

For evaluation, we assess trained agents on additional simulations across four wind configurations: ‘constant’, where the wind direction is held constant (0°) throughout the episode; ‘switch-once’, where the wind makes one 45° anticlockwise switch during the episode; ‘switch-many’, where the wind direction changes at multiple random times during the episode; ‘sparse’, which is the same as the constant configuration except that the puff birth rate is reduced (0.4-fold), resulting in more intermittent odour detections, as observed for real-world turbulent plumes. To demonstrate that our agents still perform well when odours are highly intermittent, we also include additional simulations on ‘sparser’ plumes, in which the puff radial diffusion rate is lowered (0.5-fold) in addition to lowering the puff birth rate as is done in sparse plumes. Unless otherwise specified, we describe results from one agent chosen at random from among the top five performers of 14 trained agents. See Methods and Extended Data Table 5 for more details and Supplementary Information for data on remaining agents.

### Emergent behavioural modules across varying wind conditions

**Fig. 2 |:**
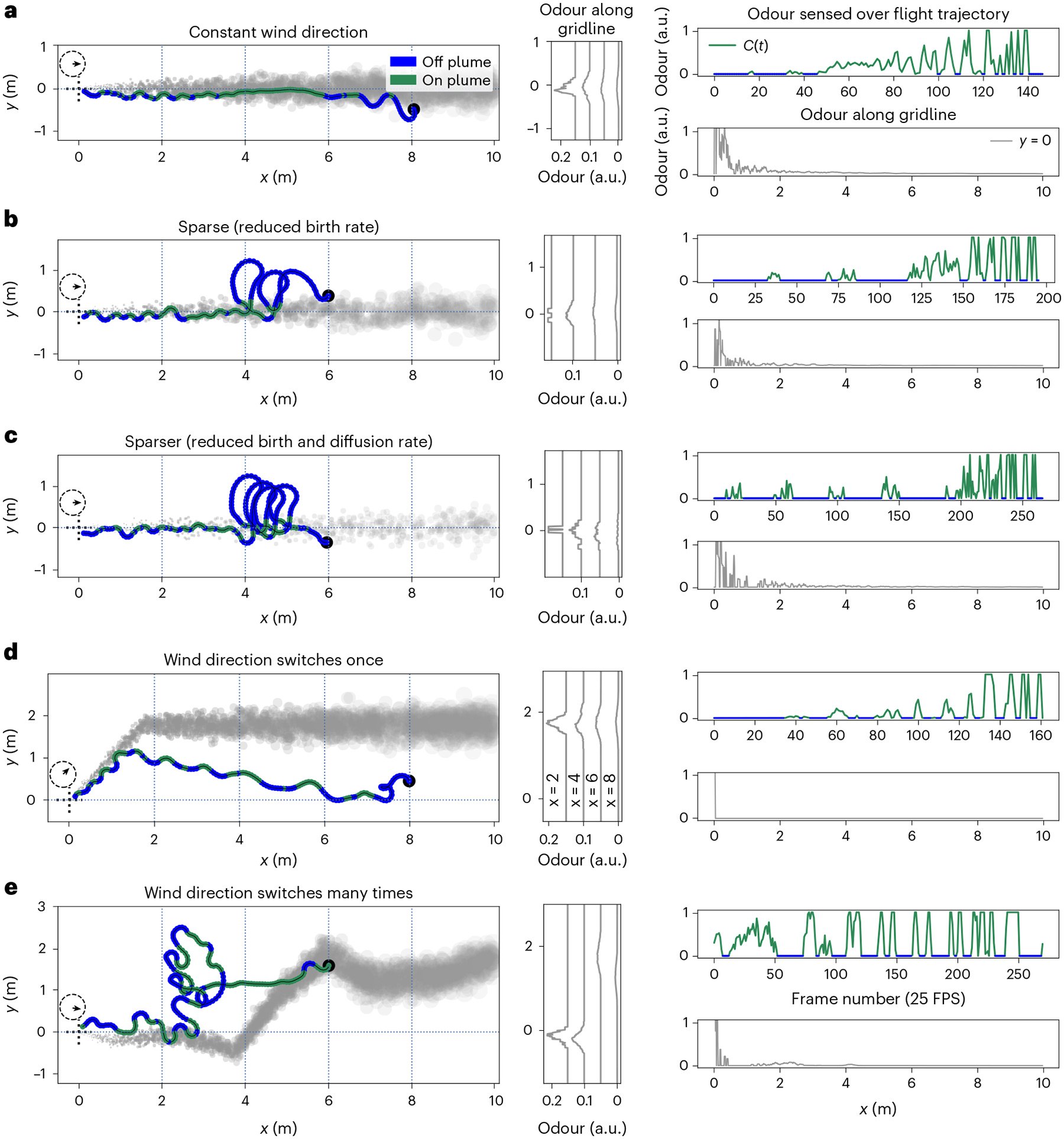
Examples of successful plume tracking trajectories and associated odour sensory streams under various plume simulator configurations. Left: snapshots of odour plumes (grey) overlaid with RNN agent trajectories, which are coloured according to whether the agent was able to sense the presence (green) or absence (dark blue) of odour. Trajectories start at the filled black circle and end at the odour source, indicated by dotted cross-hairs in the left-hand side of each panel. The plume visualizations are from the end of the tracking episode (last frame) and thus deviate from the plume as observed by the agent during the episode. The arrow within the dotted circle above the cross-hairs shows the direction of the wind at the time of the snapshot. All examples use a 0.5 m s^−1^ wind. Middle: odour concentration profiles at vertical breadthwise grid lines in the simulated arena, *x* = {2, 4, 6, 8} m. Right: odour concentration as sensed by the agent over time *C*(*t*), and odour concentration profiles along the horizontal lengthwise grid line at *y* = 0 m. **a**–**e**, Each row is a different plume configuration: constant left-to-right wind-direction plume (**a**), sparse plume with the same left-to-right constant wind direction but reduced (0.4-fold) birth rate of odour packets (**b**), sparser plume, which is like the sparse configuration and additionally has a reduced (0.5-fold) puff radial diffusion rate (**c**), switch-once plume, which makes one 45° anticlockwise wind-direction switch during the tracking episode (**d**), and switch-many plume with wind direction switches occurring every ~3 s (**e**). Animations accompanying released code provide additional examples of successful and unsuccessful tracking episodes. a.u., arbitrary units; FPS, frames per second.

**Fig. 3 |:**
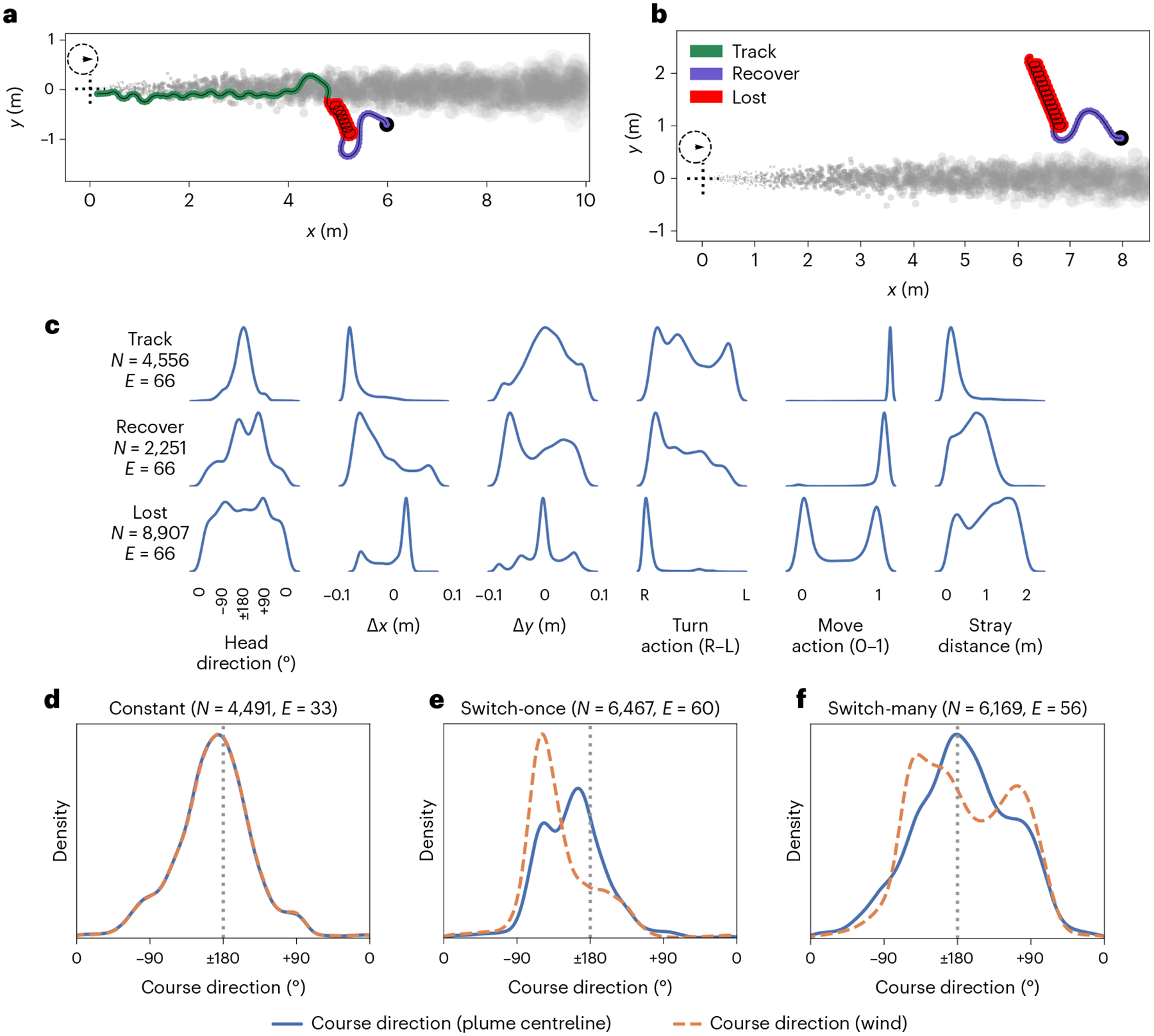
Emergent plume tracking behaviour can be decomposed into distinct behaviour modules. **a**,**b**, A successful (**a**) and an unsuccessful (**b**) plume tracking episode (RNN agent 3) showing three distinct behaviour modules: tracking (green), lost (red) and recovering (purple–blue). **c**, Kernel density estimates show data aggregated from an equal number of successful and unsuccessful constant-wind-direction plume tracking episodes (*N* time steps, *E* episodes). Plots reveal differences between the three behaviour modules across key behavioural measures: Head direction: head-direction densities are concentrated around ±180°, a signature of zig-zagging but mostly upwind movement. Angles are measured anticlockwise, with 0° indicating directly downwind. Density estimates for drift in the *x* direction (Δ*x*) and *y* direction (Δ*y*) per time step show how tracking is characterized by primarily upwind (negative *x*-direction) movement in both tracking and recover modules, but less so in the lost module. *y*-direction movements are notable in the tracking and recovering modules, corresponding to more complex turning behaviours, but minimal in the lost module. Turn action: left/right turning movements are balanced in the tracking module as the agent closely tracks the edge of the plume, but it is biased towards clockwise movements in the other two modules, especially the lost module. Move action: the agent substantially modulates its forward movement speed in the lost module only. Stray distance: the agent strays from the plume minimally in the tracking module, but substantially otherwise. Empirical distributions of course direction suggest that agents track the plume with respect to the plume centreline rather than the current wind direction. **d**–**f**, Kernel density estimates of an agent’s course direction relative to the local plume centreline (solid blue) and to the current wind direction (dashed orange) in three plume configurations. ±180° means antiparallel movement with respect to the plume centreline, or exactly upwind movement with respect to the wind direction. **d**, Constant wind configuration: the two course-direction distributions are equivalent. **e**,**f**, A substantial proportion of time is spent at an angle (≈45° angle for the switch-once configuration, **e**; arbitrary angle for the switch-many configuration, **f**) to the wind, but actually aligned (antiparallel) with the plume centreline. Bottom row panel titles indicate how many time steps and how many successful episodes were summarized in each plot.

Our trained RNN agents are able to complete the plume tracking task with changing wind direction and varying plume sparsity (Fig. 2 shows some example trajectories). The observed trajectories can be summarized by three behaviour modules, determined approximately by the time elapsed since the agent last sensed odour (Fig. 3). We refer to these three modules as ‘tracking’, ‘lost’ and ‘recovering’. In the tracking module, the agent rapidly moves closer to the plume source, using either straight-line trajectories when it is well within the plume, or a quasiperiodic ‘plume skimming’ behaviour, where it stays close to the edge of the plume while moving in and out of it. The interval between the agent’s encounters with odour packets in this module is under 0.5 s. Recovering corresponds to an irregular behaviour where the agent makes large, usually cross-wind, movements after having lost track of the plume for a relatively short period of time (about 0.5 s). Lost corresponds to a periodic behaviour that appears variably across trained agents as either a spiralling or slithering/oscillating motion, often with an additional slow drift in an arbitrary direction. This behaviour is seen when the agent has not encountered the plume for a relatively long time, typically over 1 s. Thresholds used to segment each agent’s trajectories into behaviour modules were determined by visual inspection (Extended Data Table 1).

Agents that are successful in tracking plumes in constant wind direction primarily use the tracking and recovering modules (see animations accompanying released code). Successful trajectories on the switch-once and switch-many plumes reveal that RNN agents use more complex strategies in the face of changing wind directions. If an agent is in the tracking module and well within the plume at the time of wind-direction change, it continues along its path until it reaches the edge of the plume without changing its actions. If it is skimming the edge of the plume when the wind-direction switch happens, it tries to compensate for the added movement of the plume by making more pronounced oscillations in and out of the plume. Finally, if the agent cannot keep up with the movement of the plume, it typically orchestrates a sequence of large oscillations and spiral-like movements, corresponding to the recovering and lost modules, to try to find the plume boundary. On returning to the plume, it resumes the tracking module behaviours once again.

Agents are able to execute successful tracking in sparse plumes, even when the odour encounters are increasingly intermittent (example trajectories in Fig. 2b,c). In these examples, we decreased the birth rate and diffusion rate of the odour packets in the plume simulation (Fig. 1b), resulting in environments with cross-wind odour profiles that are strongly non-Gaussian, causing even sparser odour encounters for the agent.

### Agents track plume centreline and not current wind direction

Successful trajectories in plumes that switch direction suggest that agents take the local shape of the plume into account, rather than just the current wind conditions (Fig. 3e,f and animations accompanying released code). To quantify this, we look at the empirical distributions of an agent’s course direction computed with respect to the current wind direction, and with respect to the centreline of the nearby plume (Fig. 1c). The agent’s course direction (Fig. 1f) is defined as the direction of its instantaneous movement with respect to the ground. (See Methods for details of calculations.) Figure 3 shows that the empirical course-direction distributions are much better aligned with the plume centreline than with the wind for one example agent. For switch-once plumes, the peak of the course-direction distribution is much closer to ±180° when considered relative to the centreline than relative to the wind direction. This observation indicates that the agent’s flight is on average aligned (antiparallel) with the plume centreline, but at an ≈45° angle with respect to the current wind direction. Similarly, the same trend holds in the switch-many configuration, where the course-direction distribution is aligned with the plume centreline, but diverges from the wind direction. This trend holds across all five RNN agents (Supplementary Figs. 3–12).

### Low-dimensional neural activity with task-relevant variables

We now turn our attention to the neural dynamics of the RNNs as agents perform plume tracking. Rather than characterizing the activity of individual units, we consider the population activity of the network^46^.

**Fig. 4 |:**
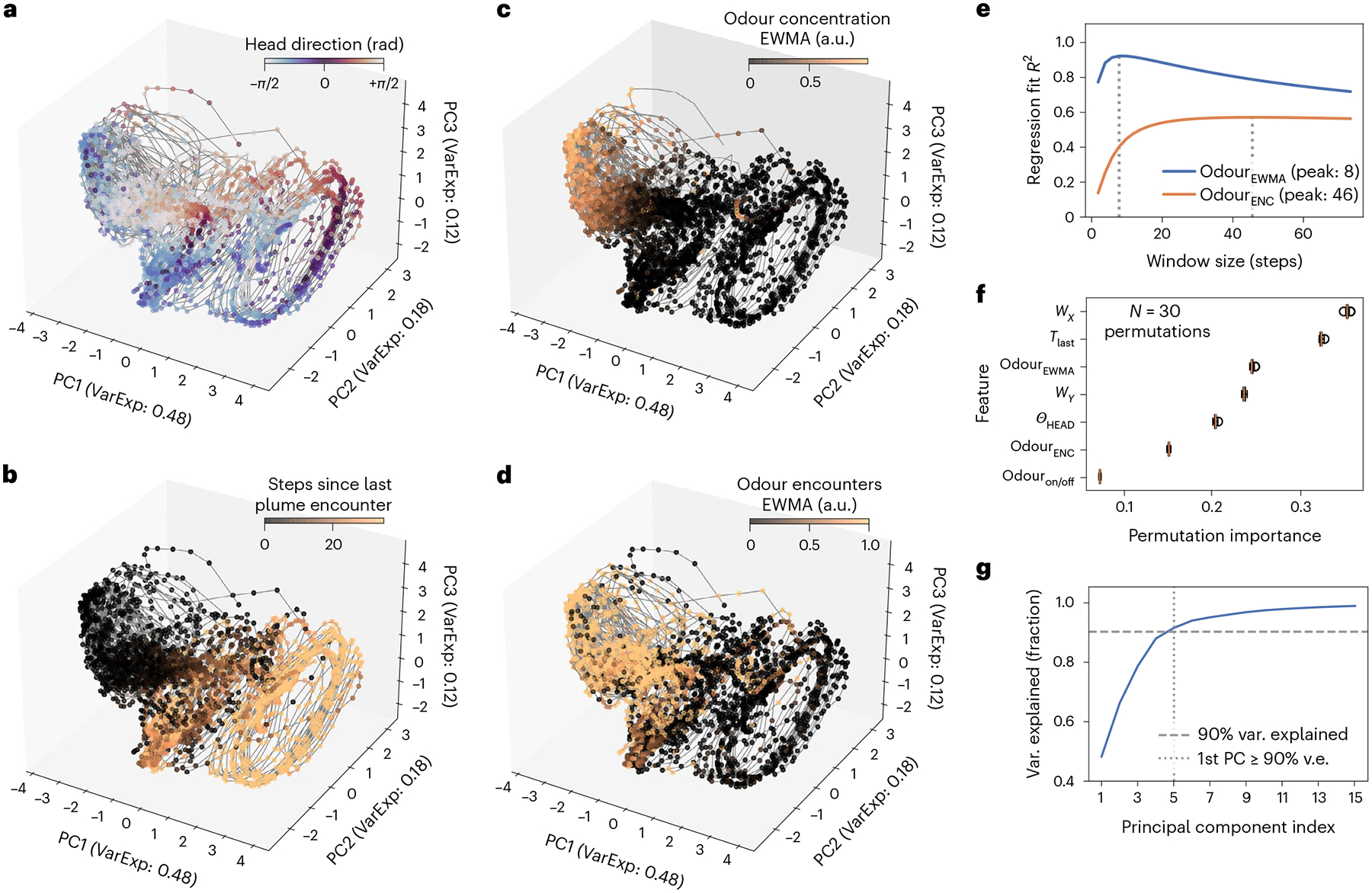
Neural activity of RNN is low dimensional and represents biologically relevant variables. **a**–**d**, Neural activity trajectories plotted over a diversity of plume conditions and tracking outcomes: **a**, coloured according to agent head direction *Θ*_HEAD_; **b**, steps since last odour encounter *T*_last_; **c**, exponentially weighted moving average (EWMA) of odour concentration (odour_EWMA_, window size 8 steps); **d**, exponentially weighted moving average of recent odour encounters (odour_ENC_, window size 46 steps). The sliding-window sizes for **c** and **d** are determined by identifying the peaks of these curves. **e**, Quality of fit (*R*^2^) of a linear model regressing neural activity onto odour_EWMA_ and odour_ENC_ for sliding windows of varying lengths. The plot of cumulative variance explained by the top principal components of neural activity aggregated across multiple plume configurations (constant, switch-once and switch-many) suggests a low-dimensional structure. **f**, Horizontal box plots of feature permutation importance scores of classifier trained to predict agent actions. Features include quantities plotted in **a**–**d** (*Θ*_HEAD_, *T*_last_, odour_EWMA_ and odour_ENC_), and instantaneous egocentric sensory observations (wind *w*_*X*_, *w*_*Y*_ and odour). Box plots show first and third quartiles (box dimensions), median (vertical line), 1.5 × interquartile range (whiskers) and outliers, if any (open circles). **g**, 90% of the variance of the 64-dimensional neural activity can be explained by the first five principal components. See Supplementary Figs. 13–17 for corresponding plots for other agents.

First, we reduce and visualize the population activity of our RNN across the constant, switch-once and switch-many plume configurations and find that the neural activity is low dimensional (Fig. 4g), with the first five to eight principal components explaining 90% of the variance in the 64-dimensional population activity. This trend holds across all five RNN agents (Supplementary Figs. 13–17).

To gain insights into the computations supporting the plume tracking behaviour, we look for variables represented in this low-dimensional population activity that are relevant for solving the task. We find that the RNNs have learned to represent task-relevant quantities beyond the instantaneous egocentric sensory observations received from the simulator (Fig. 4a–d).

Interestingly, these quantities reflect information necessary for solving these challenging plume tracking tasks and require memories of past sensory cues encountered by the agent. First, the agent’s head direction, or its orientation with respect to the ground, is evident in Fig. 4a. The time since the plume was last encountered is encoded as in Fig. 4b and may be involved in determining transitions between behaviour modules. Whereas the agent only receives local odour concentrations as a sensory input, we find that an exponentially weighted moving average of sensed odour concentrations is present in Fig. 4c. We conjecture that this quantity may be useful as a memory in the face of an intermittent odour signal arising from a patchy odour plume. Similarly, an exponentially weighted moving average of a discretized odour encounter signal is evident in Fig. 4d.

To quantify how important these represented variables are to actual task performance, we train a random forest^47^ classifier to predict the (discretized) actions taken by the agent over successful trajectories (see Methods for details). We also estimate the relative importance of each input feature by calculating its permutation importance score^47,48^, which is an estimate of the reduction in the classifier’s accuracy across several (*N* = 30) randomized permutations of that feature. Classifier accuracies using all aforementioned represented features (Fig. 4f) along with instantaneous egocentric sensory features are 10–18% higher across all agents than that using classifiers receiving just instantaneous egocentric sensory observations, and 26–51% higher across all agents than that produced by a majority-class classifier (see Extended Data Tables 2 and 3 for each agent’s feature metadata and classifier accuracies, respectively). Represented variables have permutation importance scores within the range covered by the importance scores of the instantaneous egocentric sensory inputs. Time since plume was last encountered is always one of the top two most important features, close to the *x* component of the egocentric wind velocity. The two time-averaged odour features always easily dwarf the importance of the instantaneous odour feature. Furthermore, time-averaged odour concentrations are more important than time-averaged odour encounters in four out of five agents. Head direction has an importance intermediate to the two time-averaged odour features in four out of five agents. Note that the estimates provided by this analysis are approximate due to the discretization of the action data and correlations between features.

### Neural dynamics are organized into structured regimes

**Fig. 5 |:**
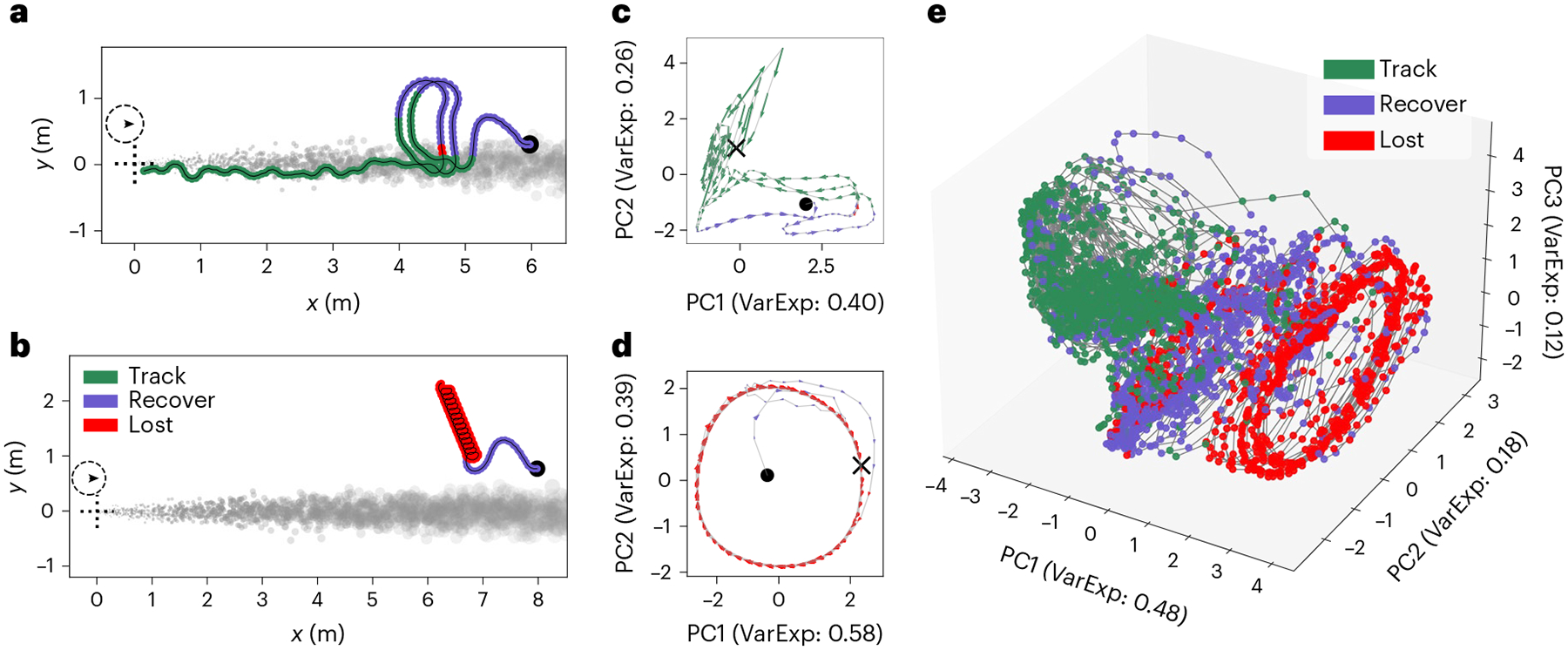
Neural dynamics appear to organize themselves into overlapping yet distinct regimes. **a**,**b**, Plume tracking episode that ends in successful homing-in on the odour source (**a**) and unsuccessful episode that strays from the plume and ends up exceeding the simulator’s bounds (**b**). **c**,**d**, Neural activity plots corresponding to each row’s trajectory projected on a two-dimensional subspace (state space) generated from the first two principal components of that episode’s neural activity. Arrows correspond to the direction of the neural activity gradient, and are coloured according to the agent’s current behaviour module. **c**, A funnel-like structure (green) emerges in the state space corresponding to the tracking behaviour module. **d**, The agent’s periodic lost behaviour shows up as a limit cycle in the state space (red). **e**, Neural activity plotted over multiple trajectories comprising a diversity of plume conditions and tracking outcomes, projected onto the first three principal components of the aggregated neural activity and coloured according to behaviour module. Examples from RNN agent 3. See Supplementary Figs. 18–22 for corresponding plots for other agents

We now examine the dynamics of the RNN activations (hidden state) and how it evolves over the course of tracking episodes. This analysis is inspired by previous work characterizing the nonlinear dynamics of RNN agents by their fixed points and transitions among them^39,41,42^. However, in a noteworthy deviation from these structures, we did not find any fixed points in our RNNs. Instead, our RNNs adopt neural dynamics that are better described by dynamical regimes. Specifically, the dynamics appear to organize themselves into overlapping but distinctly structures associated with the tracking and lost behavioural modules (Fig. 5). Interestingly, the periodic spiral or oscillatory movements seen in the lost behavioural module appear to also have a quasiperiodic limit-cycle structure in the neural state space (Fig. 5d), while the neural dynamics associated with the tracking behaviour are represented as quasiperiodic ‘funnel-like’ structures (Fig. 5c). We also see an amorphous transition region associated with the recovering behavioural module. We see the same approximate structures (limit cycles and funnel) emerge in the neural dynamics for four of the five RNN agents. See Supplementary Figs. 18–22 for data on all five agents.

### RNN connectivity reveals signatures of instability and memory

The weight matrices and recurrence Jacobians of our RNNs after training offer some theoretical insights into how the neural dynamics of the artificial agents are shaped to track plumes.

**Fig. 6 |:**
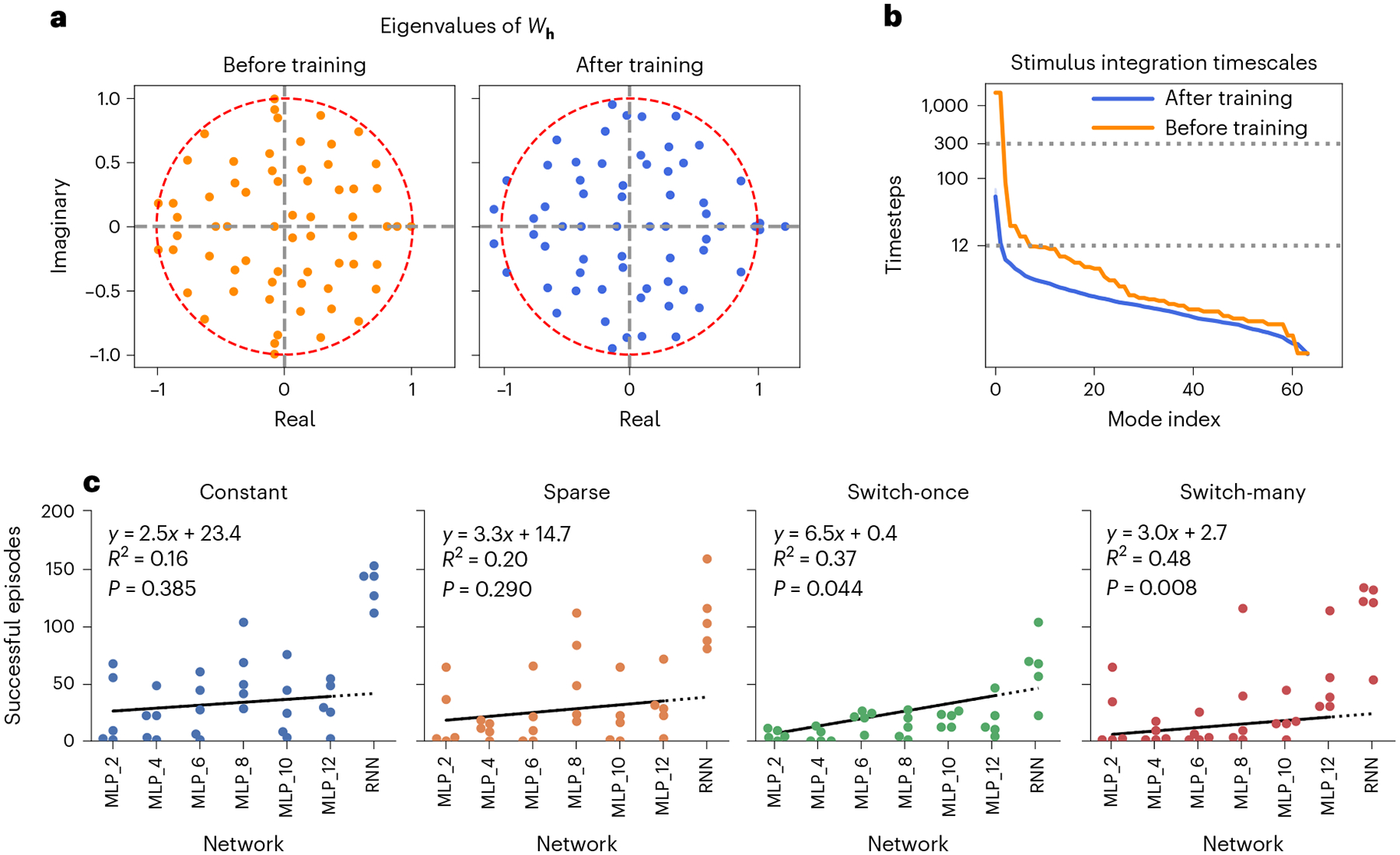
Plume tracking requires memory, especially when wind changes direction. **a**, Eigenvalue spectra of *W***h** ∈ ℝ^64×64^ (for agent 3) before and after training show how training results in the generation of unstable modes. **b**, Time-averaged (over six episodes and 1,738 time steps) stimulus integration timescales associated with stable eigenmodes of recurrence Jacobian *J*^rec^ show a bulk of relatively short timescales (within 12 time steps, lower dotted line). The top five integration timescales for the agent shown are 56.5, 13.0, 7.7, 6.8 and 5.8 time steps. Before training, timescales associated with *W*_**h**_’s eigenmodes can be large, even exceeding the length of the training/evaluation episodes (300 steps or 12 s, upper dotted line). 99% confidence interval bands have been plotted for the after-training timescale curve, but these bands are of negligible magnitude and therefore invisible. See Supplementary Figs. 23–27 for corresponding plots for other agents. **c**, Number of successful homing episodes for all five selected agents from each agent architecture, across different plume configurations for the same set of 240 initial conditions across varying agent starting location and head direction, and plume simulator state. ‘MLP_*X*’ refers to feedforward networks with *X* time steps of sensory history. Across all plume configurations, RNNs generally outperform feedforward networks, with more pronounced gains for more complex, switching wind direction (‘switch-once’, ‘switch-many’) plume tasks. In feedforward networks, performance on plumes with switching wind direction can improve statistically significantly with increasing memory. However, no statistically significant effect was observed for plumes with constant wind direction. Regression lines (solid black) are fitted on only MLP data (*N* = 30, five agents per MLP type), but are extended slightly (dotted line) for comparison with RNNs (*P* values are for a two-sided Wald test with the null hypothesis that the slope is zero).

We find that the training process reorganizes the eigenvalue spectrum of the RNN recurrence matrix *W*_**h**_ (Fig. 6a; also see Methods for definition). Before training, weights are initialized as normally distributed random variables with associated eigenvalues randomly distributed within the unit circle. After training, there are multiple eigenvalues outside the unit circle in the complex plane. Interestingly, for all five agents, there is at least one strictly real-valued eigenvalue larger than unity. Along with external stimuli, these unstable eigenvalues drive the network’s hidden dynamics.

Comparing the time-averaged stimulus integration timescales of trained RNNs (Methods) with those of the untrained RNNs reveals that training adjusts these timescales to lie well within the maximum episode length of 300 time steps (Fig. 6b). Furthermore, we see that the bulk of these timescales are within about 12 time steps (≈0.5 s), suggesting that the plume tracking task predominantly needs short-timescale memories. In Extended Data Table 4, we see that this trend holds across all five RNNs.

Finally, to understand the role of memory capacity in plume tracking, we compare the performance of our trained RNNs with trained feedforward multilayer perceptron networks (MLPs) that receive varying timescales of sensory history (Methods). As seen in Fig. 6c–f, RNNs outperform MLPs for every plume tracking task, with the performance gains being largest in the most challenging tasks. For MLPs, longer-duration sensory memories support much better performance on tougher tracking tasks, where the plumes switch more often or odour packets are sparser.

## Discussion

Our artificial RNN agents exhibit similarities to biology at the levels of behaviour, computation and neural dynamics. In this section, we draw these comparisons, discuss their significance and suggest theoretical insights that may be relevant for researchers interested in biological plume tracking.

### Behavioural features

The complex behaviour exhibited by our agents can be decomposed into simpler modules, sequenced by the time elapsed since the agent last encountered the plume (Fig. 3). These modules show features similar to upwind surging, cross-wind casting and U-turn behaviours previously reported in many studies on moths, fruit flies and other flying insects^3,5,10,49^. The spiralling behaviour seen in the agent’s lost behaviour module has been previously proposed as a plume reacquisition strategy^7^; however, it deviates from the gradually widening cross-wind casting strategy typically seen in flying insects. Furthermore, the variable sequencing behaviour modules resemble the odour-loss-activated clock mechanism that has been previously proposed to drive changes in flight behaviour in moths^50–52^.

Our observations make a behavioural hypothesis that agents track plumes with respect to the centreline rather than with respect to the current wind direction. In a previous study on tracking in constant-wind-direction plumes, ref.^53^ proposed a model where insects explicitly performed upwind surges when close to the plume centreline. However, a later study^8^ failed to find support for this model. Our analysis provides intuition for the role of centreline tracking in non-stationary plumes and suggests a testable hypothesis: we predict that centreline tracking behaviours will be more apparent in flying insects when they track plumes in wind that switches direction.

### Algorithms for odour localization

How biological organisms search and localize odour sources has a long and rich literature, and a variety of algorithms has been developed to explain this capability of single-celled organisms, cells in an organ and animals in complex environments. Where gradients exist, these smoothly varying rates of changes in concentration may be exploited to localize odour sources by chemotaxis and related algorithms^54–56^. However, in intermittent odour landscapes, gradient-based algorithms cannot be successful, and the Infotaxis algorithm was developed as an alternative^57–60^.

Both Infotaxis^58^ and our approach are formulated as solutions to plume tracking as a partially observable Markov decision process^17^. Infotaxis chooses actions (movements) to maximally reduce the expected entropy of the odour source location probability on the next time step. This makes two computational requirements of the agent. First, agents must store a probability distribution for the source location spanning the size of the arena being navigated. Second, agents must perform Bayesian inference^1^. In contrast, here our approach is to learn this control policy from only locally available measurements, and actions are chosen to maximize the expected discounted reward over a trajectory. Compared with Infotaxis, our approach produces trajectories with a stronger semblance to biology and a control policy that reacts to changing wind conditions. It also uses a neural implementation that does not make any (potentially biologically implausible) assumptions about which variables are implemented or how inference is performed.

### Neural representations

Our RNN agents learn to represent variables that have been previously reported to be crucial to odour navigation (Fig. 4). First, agent head direction has been found to be implemented as a ring attractor circuit in the central complex of many flying insects and is implicated in navigation^61–64^. Second, time since plume was last encountered is analogous to the hypothesized internal clock that determines behaviour switching in moths^50–52^. Additionally, ref.^4^ showed how this variable is encoded by the bursting olfactory receptor neurons in many animals, and that it contains information relevant to navigating in turbulent odours.

Third, the exponential moving average of odour encounters was previously^65^ found to determine the probability of turn and stop behaviours in walking flies navigating in turbulent plumes. Specifically, higher odour encounter rates were associated with more frequent saccadic upwind turns^66^. Fourth, the exponentially moving average of sensed odour concentration is motivated by previous^40^ theoretical work that posits exponentially weighted moving averages to be good canonical models for stimulus integration in RNNs. Between these two time-averaged odour variables, the best represented window length for time-averaged concentration is substantially shorter (≈0.3 s) than that for time-averaged encounters (≈1.9 s). Furthermore, we find that time-averaged odour concentration is relatively better represented and more important in predicting agent behaviour, corroborating the intuition that turn decisions during flight would require quick decision-making on subsecond timescales. We note that alternative variables beyond these four may exist that better explain agent navigation decisions.

### Neural dynamics

As often seen in neurobiological recordings^67^, the population activity of our RNNs is low dimensional, with the top five to eight principal components explaining an overwhelming majority of the 64-dimensional population’s total variance (Fig. 4g).

The neural dynamics associated with behaviour modules further exhibits interesting structure. Lost behaviours are represented as quasi-limit-cycles, while tracking behaviours show a funnel-like structure (Fig. 5). Similar one-dimensional circular manifolds and two-dimensional funnels^42,68^ have been previously reported on the representational geometry of sensory populations.

### The role of memory

Two independent analyses give us insight into the memory requirements of the plume tracking task (Fig. 6). We find that the bulk of stimulus integration timescales are within ~12 steps or 0.5 s, and that longer sensory histories and network recurrence lead to better performance on more challenging tasks, such when plumes switch direction. Together, we believe that memory is crucial for tracking plumes with non-stationary wind direction, but short timescale (under ~0.5 s) and reflexive mechanisms may be sufficient for tracking constant-wind-direction plumes. This corroborates previous results^8,53^ and extends them by highlighting the importance of longer-term memory in cases where the wind changes direction.

### Limitations and future work

Our results motivate several avenues of further development. First, our plume simulator is a computationally efficient but only approximate model that can provide a sufficiently realistic time series of odour encounters for a moving agent. However, it does not capture some aspects of real plumes, such as the filamentous nature of plumes^2^, or the variation of whiff duration and whiff frequency as a function of distance from source^69^. Further developments in efficient yet highly accurate models of turbulent flows^70^ could provide better simulations where finer-timescale interactions between agents and simulations could be learned.

Second, here we used vanilla recurrent units with no biomechanical body model, and models that incorporate known complexity from biology as constraints may give rise to further insights. For instance, DRL agents may be trained using spiking neural networks^71^. Further, the wealth of architectural insights emerging from the fly connectome may be used to constrain wiring motifs in artificial networks^72^. Modelling multiple antennae^36,73^, or more generally a biomechanical body, would enrich the interactions between the agent and the simulation environment^22,74^.

Third, multitask training should produce agents with richer behaviours and more complex neural activity structures with shared and task-specific adaptations^75,76^. Adding other sensory modalities such as vision and training the agents in a three-dimensional virtual-reality environment could produce more realistic perceptual representations in the agent^35,77^.

Finally, future work could explore learning algorithms that respect biological constraints such as excitation–inhibition balance and Dale’s law^78–80^. More complex training curricula^81^ or alternative training algorithms using evolutionary techniques^82^ might be able to mitigate the notable performance variability we observed in our agents.

Our analyses also motivate further methodological development in theoretical tools to understand actor–critic RNNs. Currently available reverse-engineering methods that characterize RNNs using discrete dynamical features such as fixed points^39–41^ are not applicable to the continuous and amorphous dynamical structures that we encountered in our analyses (Fig. 5). New methods are also needed for comparing multiple agents at the behavioural level, specifically taking into account the compounding differences that arise from small differences in action–stimulus loops. Finally, further theoretical work is required to understand the role of training-induced unstable RNN connectivity eigenmodes, such as those observed in Fig. 6, including extensions of analytic techniques developed to understand RNNs trained by supervised learning^38,40,83^.

## Conclusion

In this paper, we used DRL to train RNN agents to solve a stochastic plume tracking task. We find several behavioural and neural features that emerge in these trained agents and connect these features with how flying insects track turbulent plumes. Our findings motivate future experiments and theoretical developments, and provide a foundation for more nuanced future work. We hope our approach will contribute to the growing convergence in the understanding of artificial and biological networks^84^. Efforts to reverse engineer such neural network agents will help accelerate the development of similar methods for biological agents^85,86^. Moreover, our RNN agents may serve as generative models of complex naturalistic behaviours, which may facilitate the development of behaviour analysis tools for biology^87–89^. Insights from these studies may also inspire the development of robotic agents with artificial olfactory sensing.

## Methods

### Plume simulation

We implement a particle-based two-dimensional plume simulation model (Fig. 1f) that mimics both short-timescale features (intermittency, instantaneous concentrations) and long-timescale features (Gaussian time-averaged concentration, filamentous long-range puff transport, meandering plume structure) of real-world odour plumes evolving in a turbulent flow^43^. This type of simulation has been used in a wide range of domains including olfactory navigation^5^, robotics^92^ and sensor networks^93^. The simulator (Fig. 1b) comprises a spatially homogeneous wind vector field (0.5 m s^−1^ with configurable direction) and an odour source located at the origin that emits odour puffs as a Poisson process. Puffs are initialized with a fixed initial radius (*r*_0_) and concentration (*c*_0_). They then undergo a fixed-rate radial diffusion (*r*_*t*_ = *r*_*t*−1_ + *r*_*δ*_) such that their concentration reduces in proportion to their increase in volume, that is, *c*_*t*_ = *c*_0_(*r*_0_/*r*)^3^ *t*. In addition, each emitted puff is advected downwind at the wind velocity and perturbed randomly by cross-wind translation. In other words, each puff effectively performs a biased random walk downwind over time, while diffusing in concentration spatially. Our simulated plumes and agents are constrained to two dimensions for simplicity of analysis. The dimensions of the simulated arena are [−2 m, +10 m] and [−5 m, +5 m] in the *x* and *y* axes respectively, totalling a 120 m^2^ arena. Plumes are simulated at 100 iterations per second. The plume’s centreline is obtained by simulating puffs that have no random cross-wind translation at each iteration (Fig. 1f).

We simulate the following four wind configurations. First, the wind direction is held constant (0°) throughout the simulation (constant). Second, the wind direction makes one 45° anticlockwise switch during a tracking episode (switch-once). Third, the wind direction switches at multiple random times during a tracking episode (switch-many). Each wind direction turn is a random draw from a Gaussian distribution with mean 0 and s.d. 45°, truncated at ±60°, and occurs approximately every 3 s. Fourth, the wind direction is held constant, but the puff birth rate is reduced (0.4-fold) compared with the constant configuration (sparse). See Supplementary Section 1 for further details of the plume simulation.

### Agent architecture

Our agents are actor–critic networks (Fig. 1e), where an RNN receives sensory observations and passes a transformed representation of them onto parallel actor and critic heads that are both two-layer MLPs^44^. The actor head implements a control policy to map the RNN’s learned state representation to actions, while the critic head implements a value function that maps the state representation to an estimate of the state’s value based on rewards. This value function is used only during agent training and not thereafter. In the DRL literature, two-layer-deep heads are typically sufficiently expressive for such control problems^94^. At each time step, an agent receives a three-dimensional real-valued input vector comprising egocentric wind velocities (*x*, *y*) and odour concentration at its current location. In response, the agent produces continuous-valued turn (maximum ±6.25π rad s^−1^) and forward-movement (maximum 2.5 m s^−1^) actions; these velocities are matched to the capabilities of flying fruit flies^6,10^. In contrast to the orthogonal initialization typically employed in the mainstream machine learning literature^95^, we initialize our RNNs with normally distributed weights to facilitate comparisons with the computational neuroscience literature^75,96,97^.

Additionally, to understand the role of memory in tracking performance, we compare the RNN-based agents with an alternative feedforward-only network (MLP) architecture with fixed-length memory (Fig. 6), simulated by appending historical sensory observations onto instantaneous network inputs^98^. Although such MLPs are far from being biologically plausible architectures, they serve as useful tools for abstract comparison since their memory capacities can be controlled precisely. Both RNN and MLP layers across all agents are 64 units wide with tanh nonlinearities.

### Agent training and evaluation

We train our agents using the PPO algorithm^45^, which is known to robustly solve continuous-observation-space continuous-action-space control problems without needing substantial hyperparameter tuning. To guide agent training, we developed a curriculum and a simple reward function that greatly rewards homing in on the odour source, mildly rewards actions that reduce the radial distance between agent and odour source and penalizes longer-duration trajectories and straying too far from the plume. We train 14 independently randomly initialized networks for each architecture type, that is, RNNs and MLPs with 2, 4, 6, 8, 10 and 12 time steps of observation history.

Next, we evaluated each trained agent’s performance with a behavioural assay. Each trained agent is evaluated with 240 episodes at different initializations (15 initial locations, two initial simulation timestamps and eight initial head directions), and in each of the constant, switch-once and switch-many plume configurations. For each architecture type, we proceed to analyse only the five seeds with the best performance, as measured by total number of successful episodes across the four plume configurations. Agent training/evaluation episodes are run at 25 frames per second on a subsampled plume and limited to 300 frames/time steps (12 s of flight) per episode to accelerate DRL training. To demonstrate agent performance on more patchy odour plumes, the simulations used for Fig. 2c (and all analyses in Supplementary Section 7) use a plume radial diffusion rate that is 50% of the rate used while training. See Extended Data Table 5 for all associated hyperparameters, and Supplementary Section 1 for additional details on agent training and evaluation.

### Agents track plume centreline and not current wind direction

Subtracting the current wind-direction angle from the course direction provides the course direction with respect to the wind. To find the course direction with respect to the centreline, we first find the median local centreline angle using centreline puffs (Fig. 1c) within a ±2 cm band of the *x* coordinate of the agent’s location, then subtract this from the course direction with respect to the ground. The empirical distributions include aggregate data from when agents are in the tracking behaviour module from up to 60 random successful trajectories from the constant, switch-once and switch-many plume configurations. Additionally, for the switch-once configuration, we trim trajectories to consider only the time steps after the wind-direction switch has occurred.

### Neural activity dimensionality and neural representations

#### Odour encounters.

Our definition of odour encounters is identical to that used by Demir et al.^65^ The stream of odour inputs is discretized to be 1 at the first time step of the stream where the odour is perceptible and 0 for the remaining contiguous steps where it is still perceptible.

#### Agent action classifier.

To quantify how important these represented variables are to actual task performance, we train a random forest^47^ classifier to predict actions taken by the agent over successful trajectories. We uniformly partition the turn and move action variables, which are continuous valued, into domains of three and two discrete classes respectively. These classes correspond roughly to ‘left’, ‘centre’ and ‘right’ turns, and to ‘fast’ and ‘slow’ forward movements. These are concatenated to form a six-class independent variable. The classifier receives instantaneous sensory observations (egocentric wind speed *x* and *y* components *w*_*X*_, *w*_*Y*_ and odour concentration) and the four aforementioned encoded features as inputs. Training and test sets are a randomized non-overlapping 80%–20% split of evaluation episodes, balanced across plume configuration and episode outcomes. We make a 20-trial threefold cross-validated randomized search over the number-of-estimators (range [10, 50]) hyperparameter, and then train a classifier using the best hyperparameter on the whole training set. We next estimate the relative importance of each input feature by calculating its permutation importance score^47,48^, which is an estimate of the reduction in the classifier’s accuracy across several (*N* = 30) randomized permutations of that feature. Note again that the estimates provided by this analysis are approximate due to the discretization of the action data and correlations between features.

We determine the window sizes^99^ for odour concentrations and encounters by linearly regressing neural activity onto them for sliding windows of varying lengths, and we choose the window size that produces the best fit as measured by the coefficient of determination *R*^2^ (Fig. 4e). The best moving-average window length for time-averaged odour concentrations (seven time steps or 0.3 s on average across all five agents) is substantially shorter than that for time-averaged odour encounters (47 time steps or 1.9 s on average across all five agents). Time-averaged odour concentrations are also better encoded (*R*^2^ = 0.91 on average across five agents) than time-averaged odour encounters (*R*^2^ = 0.59 on average across five agents). See Extended Data Table 2 for data on each of the five RNN agents.

### RNN connectivity analysis

The update rule for a vanilla RNN with hidden state vector **h**_*t*_ is given by

$$h_{t}=F\left(h_{t-1},x_{t}\right)=\text{tanh}\left(W_{h}h_{t-1}+W_{x}x_{t}+b\right),$$

where *W*_**h**_ is the recurrence (connectivity) matrix of the hidden layer, **x**_*t*_ are the network’s inputs, *W*_**x**_ is the input-to-hidden layer matrix and *b* is a bias term^39^. Next, we consider a linearization of this nonlinear system around arbitrary expansion points. The RNN update equation can be linearized around an arbitrary expansion point (**h**^e^, **x**^e^) to obtain a linear dynamical system approximated by

$$h_{t}\approx F\left(h^{\text{e}},x^{\text{e}}\right)+J^{\text{rec}}|{\;}_{\left(h^{\text{e}},x^{\text{e}}\right)}\Delta h_{t-1}+{J^{\text{inp}}|}_{\left(h^{\text{e}},x^{\text{e}}\right)}\Delta x_{t},$$

where Δ**h**_*t*−1_ = **h**_*t*−1_ − **h**^e^ is the state of the linearized system, Δ**x**_*t*_ = **x**_*t*_ − **x**^e^ is the linearized system’s input and *J*^inp^ is the input Jacobian^40^. To be explicit,

$${J_{ij}^{\text{rec}}|}_{\left(h^{\text{e}},x^{\text{e}}\right)}=\frac{\partial F{\left(h,x\right)}_{i}}{\partial h_{j}},$$

$${J_{ij}^{\text{inp}}|}_{\left(h^{\text{e}},x^{\text{e}}\right)}=\frac{\partial F{\left(h,x\right)}_{i}}{\partial x_{j}}.$$

Note that *J*^rec^|_(0,0)_ = *W*_**h**_ and *J*^inp^|_(0,0)_ = *W*_**x**_.

Previous literature has looked at the eigenvalues and eigenvectors of the recurrence Jacobian (and recurrence matrix) to investigate how connectivity affects the dynamics of the network^38,40^. Specifically, Maheswaranathan et al.^40^ obtain the stimulus integration timescale *τ*_*i*_ associated with a stable eigenvalue *λ*_*i*_ (that is, ∣*λ*_*i*_∣ ≤ 1) by looking at the discrete-time iteration $h_{i}(t)=\lambda_{i}^{t}h_{i}(0)$ that governs the integration of stimulus in the direction of eigenvector **v**_*i*_ associated with *λ*_*i*_. They then compare this with the equivalent continuous time equation $h_{i}(t)=h_{i}(0){\text{e}}^{-t/\tau_{i}}$ to obtain *τ*_*i*_ = |(1/ln|*λ*_*i*_|)|. Following their approach, we consider the eigenvalues of the recurrence Jacobian and associated stimulus integration timescales along the trajectories of several episodes. This timescale governs the integration of stimuli in the direction of the corresponding eigenvectors. We chose at random one successful and one unsuccessful episode from each of three plume configurations (constant, switch-once and switch-many). At each time step of the trajectory, we computed the recurrence Jacobian assuming zero input *J*^rec^|_(**h**,0)_.

## Extended Data

**Extended Data Table 1 |:** Thresholds for defining when the lost behaviour module kicks in that is duration (in timesteps or seconds) since the plume was last encountered

Agent	Agent ID	Lost module threshold
RNN 1	2760377	30 steps (1.2 s)
RNN 2	3199993	25 steps (1.0 s)
RNN 3	3307e9	35 steps (1.4 s)
RNN 4	541058	38 steps (1.52 s)
RNN 5	9781ba	25 steps (1.0 s)

**Extended Data Table 2 |:** Moving window lengths and linear regression fit R_2_ for two represented variables: odor_EWMA_ and odor_ENC_. (Recall that 25 timesteps = 1.0 second)

Agent	Agent ID	*odor*_*EW MA*_ window [steps]	*odor*_*EW MA*_ *R*^2^	*odor*_*ENC*_ window [steps]	*odor*_*ENC*_ *R*^2^
RNN 1	2760377	8	0.91	62	0.57
RNN 2	3199993	10	0.86	44	0.71
RNN 3	3307e9	8	0.92	46	0.57
RNN 4	541058	6	0.88	40	0.51
RNN 5	9781ba	12	0.91	44	0.59

**Extended Data Table 3 |:** Classifier based quantification of contribution of represented features. In last two columns, quantity in parentheses is the difference in accuracy with respect to classifier that has all features (4 represented features and instantaneous egocentric sensory features). Represented features contribute to higher test accuracy

Agent	Agent ID	Test set accuracy (All features)	Test set accuracy (Instantaneous only)	Test set accuracy (Most freq. class)
RNN 1	2760377	0.84	0.74 (0.10)	0.33 (0.51)
RNN 2	3199993	0.67	0.49 (0.18)	0.28 (0.39)
RNN 3	3307e9	0.82	0.69 (0.13)	0.39 (0.43)
RNN 4	541058	0.70	0.53 (0.17)	0.44 (0.26)
RNN 5	9781ba	0.84	0.74 (0.10)	0.40 (0.44)

**Extended Data Table 4 |:** Top 5 *τ*s (stimulus integration timescales) for each RNN seed

Agent	Agent ID	Top 5 *τ*s
RNN 1	2760377	116.5, 81.5, 16.9, 13.5, 8.3
RNN 2	3199993	95.7, 61.7, 16.6, 12.0, 9.6
RNN 3	3307e9	56.5, 13.0, 7.7, 6.8, 5.8
RNN 4	541058	86.4, 51.8, 15.1, 12.4, 9.7
RNN 5	9781ba	86.2, 27.4, 8.6, 6.6, 5.6

**Extended Data Table 5 |:** Parameters for simulator, environment, agent/model, and training

Parameter description	Value/Range
Simulation integration time-step	0.01s
Simulation wind speed	0.5 m/s
Simulation wind speed crosswind noise	𝒩(0,0.005) m/s (per timestep)
Simulation puff birth rate (Poisson mean)	1.0 puffs/timestep (at 100 FPS)
Simulation puff initial radius	0.01m
Simulation puff radius growth rate (radial diffusion-rate)	0.01m/s (= 1.0x)
Simulation maximum plume extent simulated (x, y)	(−2/+10m, ± 5m)
Environment frame rate	25 FPS
Agent sensor sampling rate	25 Hz
Agent forward movement capacity (Δ_*max*_)	2.5 m/s
Agent turn capacity (*θ*_*max*_)	± 6.25 π radians/s (± 1125 °/sec)
Homing radius	0.2 m
Maximum stray from plume allowed	2 m
Agent odor sensing thresholds (minimum, maximum)	(0.0001, 1.0) (A.U).
RNN hidden layer width	64 units
Feedforward hidden layer width(s)	64 units
Model nonlinearity	tanh
Model layer initializations (Recurrent, Feedforward)	(Normal, Orthogonal)
RNN training steps	5M
MLP training steps	2M
Learning Rate	0.0003 (with linear decay)
Proximal Policy Optimization (PPO) Entropy Coefficient	0.05
PPO Value Loss Coefficient	0.5
PPO Epochs	10
PPO Gamma	0.99
PPO maximum gradient norm	0.5
Generalized Advantage Estimation (GAE) Lambda	0.95
GAE steps	2048

## Supplementary Material

Supplementary Material

## Data Availability

The datasets generated during and analysed during the current study are publicly available in the accompanying figshare repository^100^.
