# Supplementary material for: Emergent behaviour and neural dynamics in artificial agents tracking odour plumes
Source: Nat Mach Intell. Author manuscript; Available in PMC 2023 Oct 26. (PMC10601839; doi:10.1038/s42256-022-00599-w)
Supplement: Supplementary Material [file NIHMS1932434-supplement-Supplementary_Material.pdf]

nature machine intelligence

Article https://doi.org/10.1038/s42256-022-00599-w

Emergent behaviour and neural dynamics in artificial agents tracking odour plumes

In the format provided by the authors and unedited

SUPPLEMENTARY MATERIAL

We repeat some details from the main text for the sake of readability.

SI1 Supplementary details on agent training and evaluation

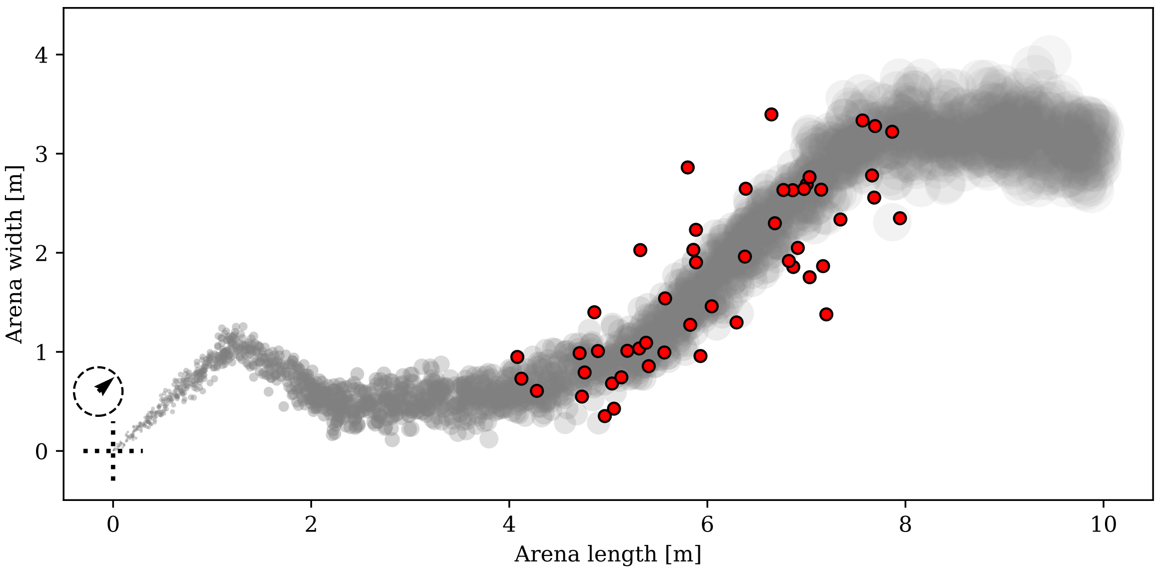

Figure SI1: **Snapshot of training plume:** Plume (dull grey) originating at crosshairs (dotted black lines, bottom left). Current wind direction shown by arrow in dashed circle (bottom left). 50 randomly chosen initialization points (red) overlayed on plume. Agent is initialized with a uniformly randomly chosen head-direction at a random location near or on the plume. Wind direction switches by a random amount at random times as described in Section SI1

Plume pre-computation:

Puffs are generated at the source located at $(0,0)$, at the rate of $r_t \sim Poisson(R)$ puffs/step, where $R = 1.0$. Each puff's location $p_t = (x_t, y_t)$ is henceforth governed by the stochastic differential equation, $p_t = p_{t-1} + w_t\delta + \xi$, where w_t is the wind-velocity at time t, $\xi \sim \mathcal{N}(0,\sigma)$ is cross wind i.i.d. random Gaussian noise added per the odor plume model of Farrell et al.[43] Each puff trajectory is integrated using a simple forward Euler integrator at 100 frames/sec. Furthermore, each puff starts with a radius $r_0 = 0.01$ m and undergoes a diffusion process that increases it's radius at the rate of 0.01 m/s. Puff concentrations reduce in proportion to their increase in volume due to the increased radius (see Methods for formula).

We compute 120-second long (clock time) plumes ahead of training/evaluation time for the 'constant', 'switch-once' and 'switch-many' configurations. A 40s window (60s - 100s) of the 'switch-many' and 'constant' plumes are used for training agents (Figure SI1). 'sparse' plumes are simulated by downsampling the number of puffs simulated in a 'constant' plume simulation. 'sparser' plumes are 'sparse' plumes with reduced (0.5x) radial diffusion-rates to encourage more *patchy* plume landscapes.

Wind velocity w_t is held constant at $(0.5, 0.0)$ m/s for all t for the 'constant' plume configuration. For the 'switch-once' plume configuration, wind velocity w_t starts at $(0.5, 0.0)$ m/s till $t = 60.00$ s, when it makes a single 45° counter-clockwise turn and stays there for the rest of the simulation. For the 'switch-many' plume configuration, wind velocity w_t changes once every $3.0 + \tau$ seconds, where $\tau \sim Uniform(-0.3, +0.3)$ is a random shift added i.i.d. at each change. wind direction turns are sampled i.i.d. from a $\mathcal{N}(0°, 30°)$ Gaussian distribution truncated at ±60°.

Training

Partially Observable Markov Decision Process (POMDP): To train agents using DRL, we define a Partially Observable Markov Decision Process (POMDP)[17] as follows (also see Figure 1). While a belief-state is not explicitly defined, we use an RNN (or MLP with past inputs) to learn and estimate a belief-state-like representation from the history of past observations. Although an explicit representation of uncertainty is not computed in our model, the RNN state representation, when combined with reinforcement learning, allows the model to approximate a solution to the underlying POMDP problem.

- *Action space:* Agents provide a two dimensional output a_t at each timestep corresponding to how much they want to turn and how much they want to move forward.

$$\mathbf{a_t} = [a_\theta, a_m], \text{where } a_\theta \in [-\theta_{max}, +\theta_{max}], a_m \in [0, \Delta_{max}]$$

  The maximum turn capacity of an agent (θ_{max}) is 6.25π radians/s (1125 °/s), and the maximum forward movement capacity (Δ_{max}) of an agent is 2.5 m/s.

- *Observation space:* Agents receive a 3-dimensional egocentric sensory observation vector o_t at timestep t, comprising odor-concentration and (x, y) components of relative wind-velocity at the agent's current location and orientation in the plume. Note that the agent's current location and orientation in the plume are tracked and updated by the training environment code.

$$\mathbf{o_t} = [o_c, o_x, o_y], \text{where } o_c \in [c_{min}, c_{max}], o_x, o_y \in [-(\Delta_{max} + |v_{wind}|), (\Delta_{max} + |v_{wind}|)]$$

  Here c_{min} and c_{max} are the minimum and maximum perceivable odor concentrations, that have been manually set to be 0.0001 and 1.0 arbitrary units respectively.

- *Reward function::* Rewards are given to encourage task completion, i.e. home in on the plume source. The agent receives: +100 when it reaches within a small fixed radius $r_{homed} = 0.2m$ of the source, $-\epsilon$ per timestep to simulate a 'metabolic cost' to flying and therefore encourage faster homing. We also provide the agent two shaping rewards, without which the training process is infeasibly slow: First, a reward proportional to the decrease in radial distance to the source per timestep $(r_{t-1} - r_t)$ as a form of shaping reward. Here, $r_t = \sqrt{x_t^2 + y_t^2}$ is the euclidean distance of the agent to the source at timestep t. Second, a fixed negative reward of -10 if the agent strays more than $r_{stray} = 2$m away from the plume (i.e. the center of the nearest puff is greater than r_{stray}).

- *Transition function:* The agent's location and orientation within the arena is randomly initialized at the beginning of each training episode (see Figure SI1 for example locations). The environment then deterministically updates the agent's location and orientation at each timestep taking into account its actions and the wind velocity. Episodes end if the agent reaches within a radial distance r_{homed} of odor source, or if the agent strays more than r_{stray} from the plume, or if the episode exceeds 300 timesteps (12 seconds of clock time).

- *Augmented observation space for MLPs:* To understand the role of memory on tracking performance (Ref. Figure 6), we use feedforward-only networks (MLPs) with fixed-length memory. Memory is simulated by appending historical sensory observations into the MLPs' inputs (known as 'frame stacking' in the DRL literature[96]). Therefore $\mathbf{o_t}$ for an MLP with L timesteps history is now $[o_c^{(0)}, o_x^{(0)}, o_y^{(0)}, \ldots o_c^{(L)}, o_x^{(L)}, o_y^{(L)}]$.

Observations are received from the environment, processed and acted upon by the agents at each timestep (25 FPS or every 40ms). We implement the POMDP environment using the OpenAI Gym[102] and stable-baselines[92] libraries.

Training curricula: We adapt an open source implementation[101] of the Proximal Policy Gradient algorithm with Generalized Advantage Estimation (PPO-GAE)[103,45] to train our agents.

To train our agents to perform across dynamically varying plumes, we randomize the agent's location, agent's orientation, plume state and plume sparsity at the start of each training episode. Agents are initialized at random starting locations (x, y), where x is chosen uniformly randomly in the range $[30, 80]$ percentile of puff locations; y is chosen by sampling from a normal distribution with mean given by the median y-coordinate of odor puffs in the range $[x-1, x+1]$, and variance given by the $5^{th} - 50^{th}$ percentile y-coordinate difference of the aforementioned odor puffs. Initial agent orientation is selected at random from $[-\pi, \pi]$ radians. The 'switch-many' plume, which changes direction every $\approx$ 3 seconds, is used for training. Initial plume state is randomized by choosing a random time between 60s - 90s, at which to initialize the precomputed plume. The simulation is sparsified by downsampling the number of puffs to a fraction randomly uniformly chosen in the range $[0.3, 1.0]$. The plume is randomly flipped about the x-axis to mitigate any y-directional biases that might have crept into the finite plume simulation.

Curriculum based training methods are known to improve training performance by gradually increasing the difficulty of the training task over the course of the training process.[81] We train our RNNs using a two stage curriculum, where we first train the RNN for 1 million timesteps on the constant wind direction plume, and then train it for another 4 million timesteps on the 'switch-many' plume. This two stage process improved the stability and performance of the training process for RNNs, but not for MLPs. MLPs are directly trained for 2 million timesteps on the 'switch-many' plume. Training durations have been chosen such that training updates reliably converge within these times.

Hyperparameter selection: Our training process has hyperparameters relating to (1) training algorithm hyperparameters, (2) training plume parameters, and (3) neural network architecture. (See Extended Data Table 5 for a list of all [hyper]parameters and values). While PPO is not the most sample efficient algorithm, it is known to work robustly across a wide range of continuous control (continuous observation and action space) problems without needing extensive hyperparameter tuning.[45,104] Furthermore, exhaustive hyperparameter tuning is computationally unfeasible on our budget. However, we do try to tweak hyperparameters one-by-one starting off from the parameters suggested in the PPO manuscript for continuous control problems.

To decide network width, we trained RNNs with 24, 32, 64, 96, 128 and 256 units, and found no improvement in performance by increasing width beyond 64 units. We found that training for networks with 32 or 24 units converged less often and their test performance was lower than wider networks. This is expected because there's often a "minimum capacity" of network required to easily train a network for a task; see, for example, the simple benchmark tasks (with 3-dimensional inputs) and networks used in Maheswaranathan et al.[41]

For RNN-MLP comparisons, since we are trying to artificially limit the amount of memory available to the agent, we chose to vary the "input half" of the network, and keep the architecture of the actor and critic sub-networks unchanged. To do this, we had to keep the RNN and MLP layer widths identical. We believe that this makes the comparison between RNNs and MLPs fairer since the state representation dimension (i.e. the RNN hidden state dimension, or the MLP intermediate layer width) remains the

same across networks. Finally, we also trained Gated Recurrent Units (GRUs) in the same manner as we did our Vanilla RNNs (RNNs), and found that the performance of the GRUs did not significantly exceed that of the RNNs (see Figure SI2).

For MLPs, we limit ourselves to 12 timesteps of memory (historical observations) because we observe that MLP performance improvement plateaus around 10 to 14 timesteps of memory, and then reduces as we increase memory. It is known that the optimization problem grows exponentially harder with increase in the size of the input space. RNNs do not require any history stacking (and store memory in the form of internal state) and are therefore preferred over MLPs for precisely this reason when the problem involves long-timescales.

Other shaping rewards explored: Flying insects are known to exhibit a significant range of speeds.[10] However, our trained agents mostly fly at either their maximum speed or very slowly (see Figure 3). As additional reward shaping, we did try to add movement-related penalties to the reward function to induce some speed modulation, however, did not use these agents because of drastically worse performance compared to unpenalized agents. Future work could explore ways of skewing DRL reward functions towards such auxiliary goals that are not aligned with the primary plume tracking task.

Computational resources: All models are trained and evaluated on an Ubuntu Linux v20.04 workstation with Intel Core i9-9940X CPU and a TITAN RTX GPU. Each seed takes ≈ 16 hours to train and evaluate, with MLP and RNN models using 1 and 4 cores in parallel respectively.

Evaluation

We evaluated trained agents over a behavioral assay comprising fixed set of initial locations, initial simulation timestamps and initial agent directions across the aforementioned plume wind direction and birth-rate configurations, each comprising 240 episodes.

The same set of 240 initial conditions for each episode are used to initialize the agent and simulator, for each agent and dataset evaluated:

- Initial agent head angle (with respect to ground): $0, \frac{1}{4}\pi, \frac{1}{2}\pi, \frac{3}{4}\pi, \pi, \frac{5}{4}\pi, \frac{3}{2}\pi, \frac{7}{4}\pi$ radians
- Initial x-coordinate: 4, 6, and 8 meters
- Initial y-coordinate: 0th, 25th, 50th, 75th, and 100th percentile of the minimum and maximum y-coordinate of the puffs located in a 1-meter band around the initial x-coordinate. For 'constant' wind direction plumes (including sparse plumes), the task is made harder by selecting only 0th, 50th, and 100th percentiles as described before (i.e. $y_{min}, y_{median}, y_{max}$) and then adding two other locations that are ±0.5 m outside the plume (i.e. $y_{min} - 0.5$ m and $y_{max} + 0.5$ m)]
- Initial timestamp: 60.00s and 61.00s (58.00s and 59.00s for the 'switch-once' plume as it switches at exactly 60.00s)

Agent selection: We train 14 seeds per model type (RNNs, and MLPs with 2, 4, 6 ..., 12 timesteps of history) and select the top-5 best performing seeds for analysis. Performance here is measured by counting the number of successful episodes across 'constant', 'switch-once' and 'switch-many' plumes.

Evaluation subset: For all our analyses in Results, we use a randomly selected 120 episode *evaluation subset* of the 240 evaluation episodes for each of the constant, switch-once and switch-many plume configurations. The selected episodes are balanced to include an equal number (60 episodes each) of successful and unsuccessful plume tracking episode outcomes. Whenever there are fewer than 60 episodes of either outcome type (successful or unsuccessful) for any plume configuration, then the selection is trimmed to use an equal number of episodes of the smaller outcome type. PCAs tend to be sensitive to imbalances in the data, and this balancing process enables visualizations to be consistently compared across agents. In the analysis described in Figure 6c-e, we use all 240 evaluation episodes per agent.

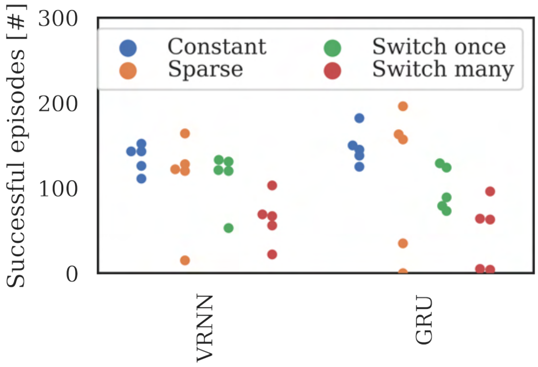

Figure SI2: Comparison of Vanilla RNNs and GRUs across 4 plume configurations. Vanilla RNN data is same as that in Figure 6
.

SI2 Behavior module metadata distributions

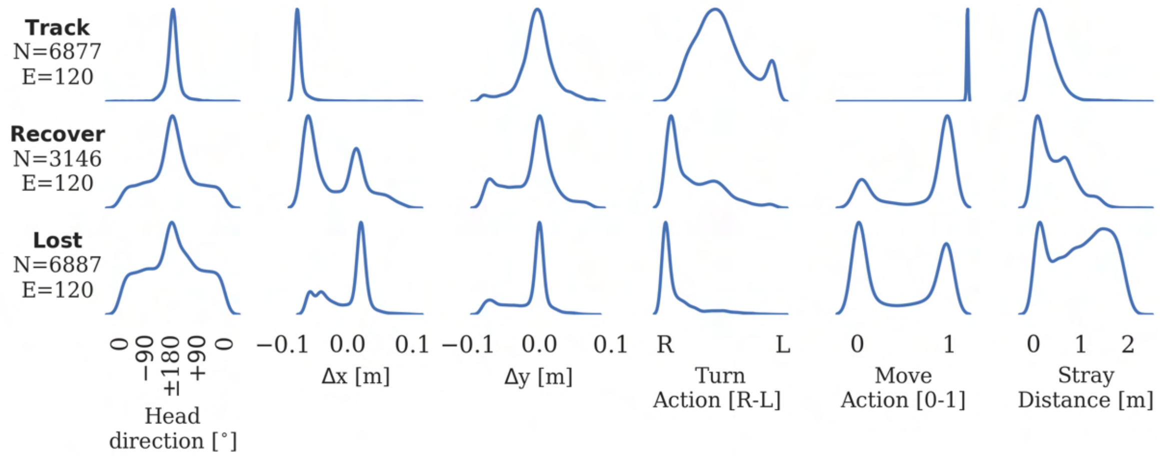

Figure SI3: Behavior modules - Agent 1 (See Figure 3 for equivalent data on Agent 3 and figure details)

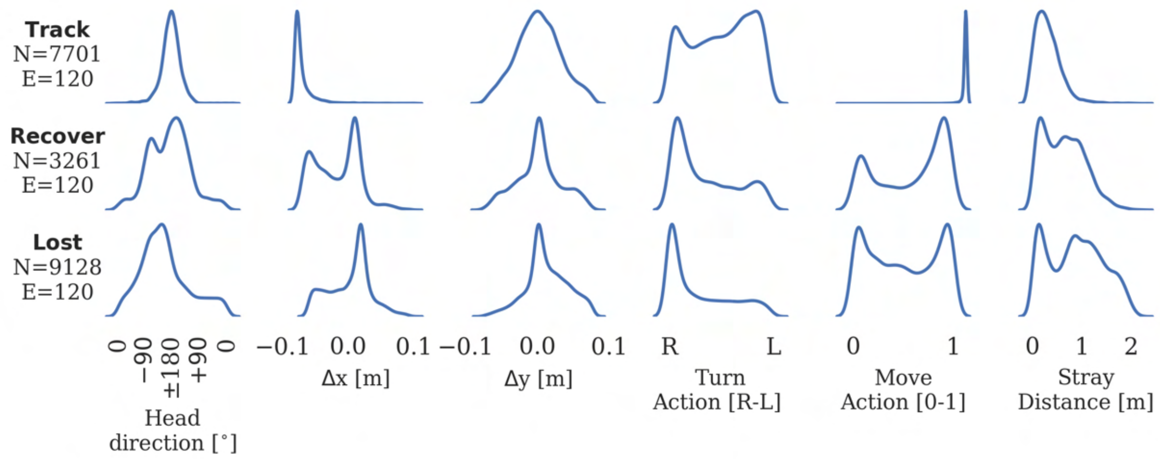

Figure SI4: Behavior modules - Agent 2

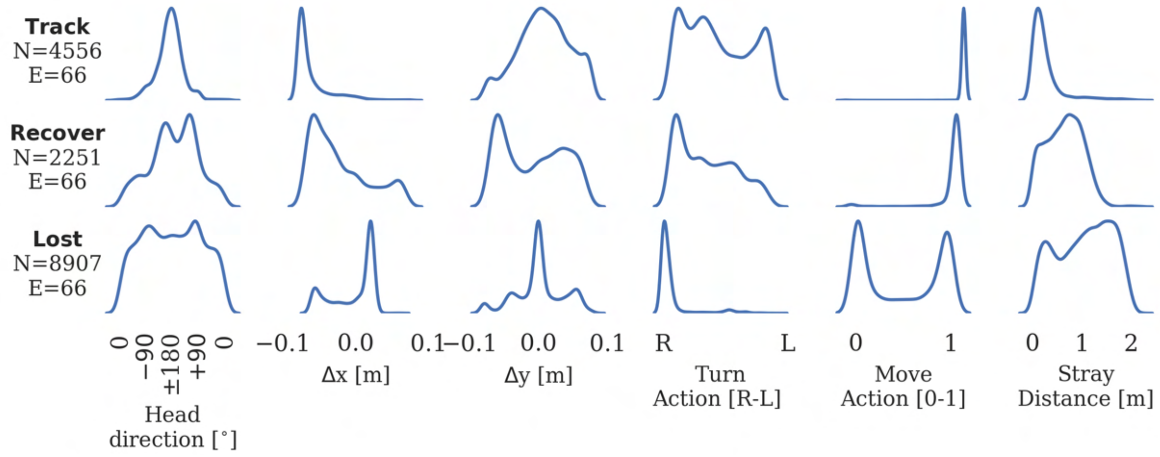

Figure SI5: Behavior modules - Agent 3 (same as Figure 3)

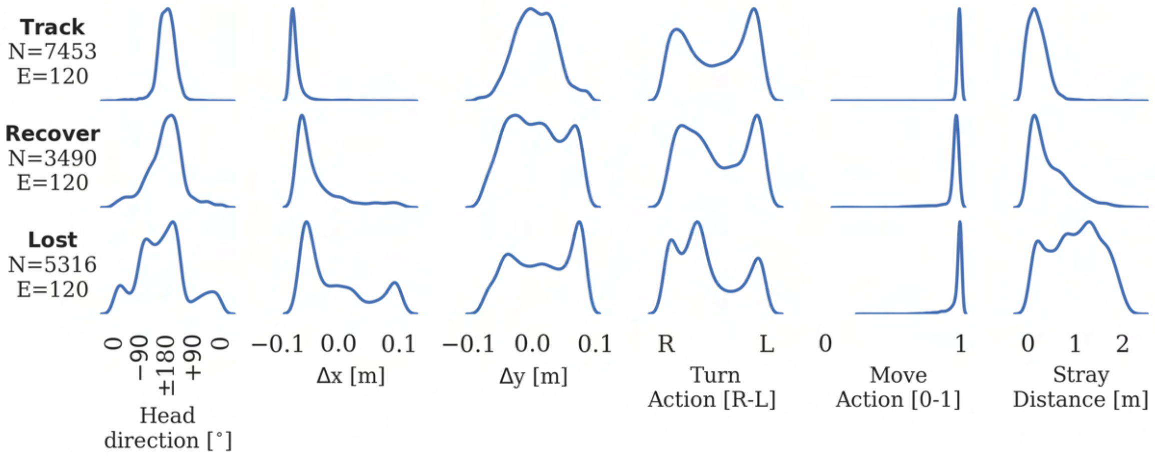

Figure SI6: Behavior modules - Agent 4

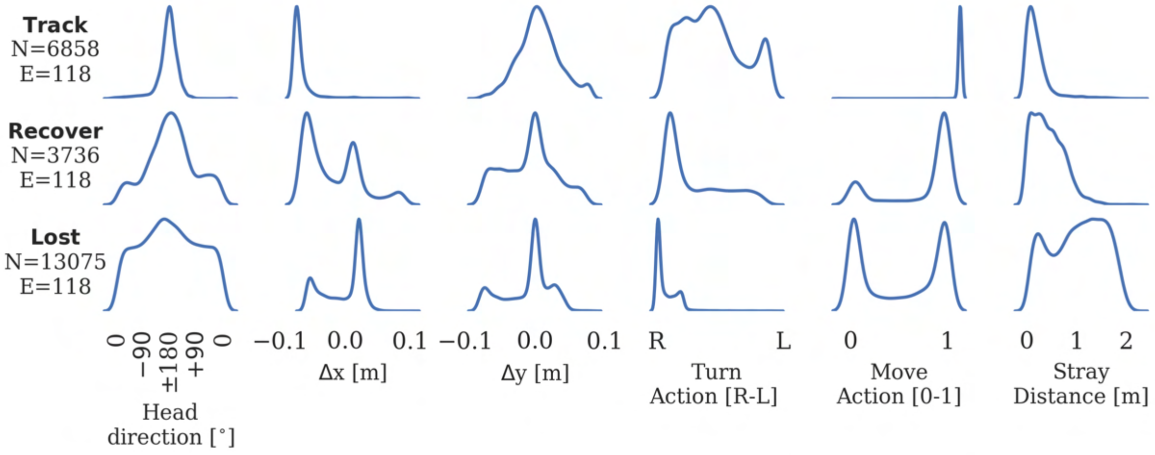

Figure SI7: Behavior modules - Agent 5

SI3 Agents track plume centerline, not current wind direction

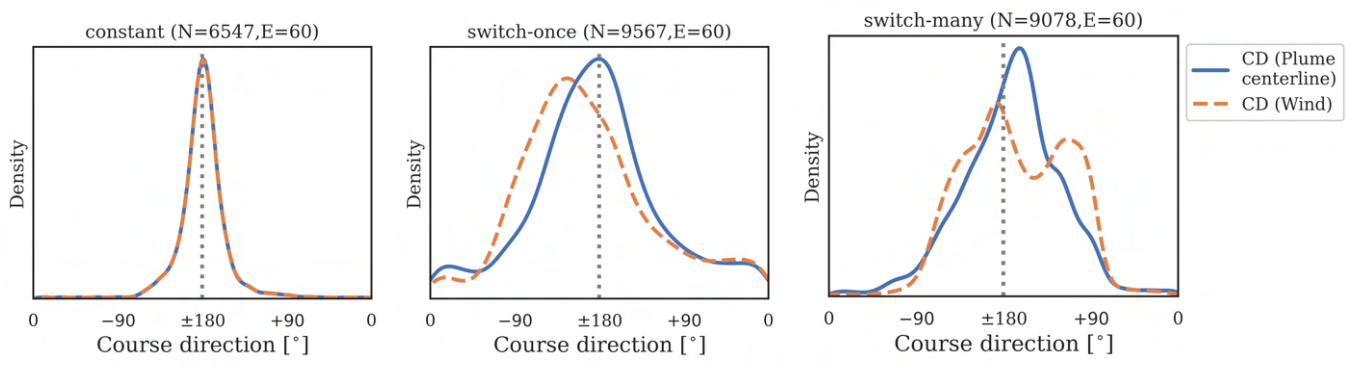

Figure SI8: Empirical course-direction (CD) distribution - Agent 1 (See Figure 3 for equivalent data on Agent 3 and figure details)

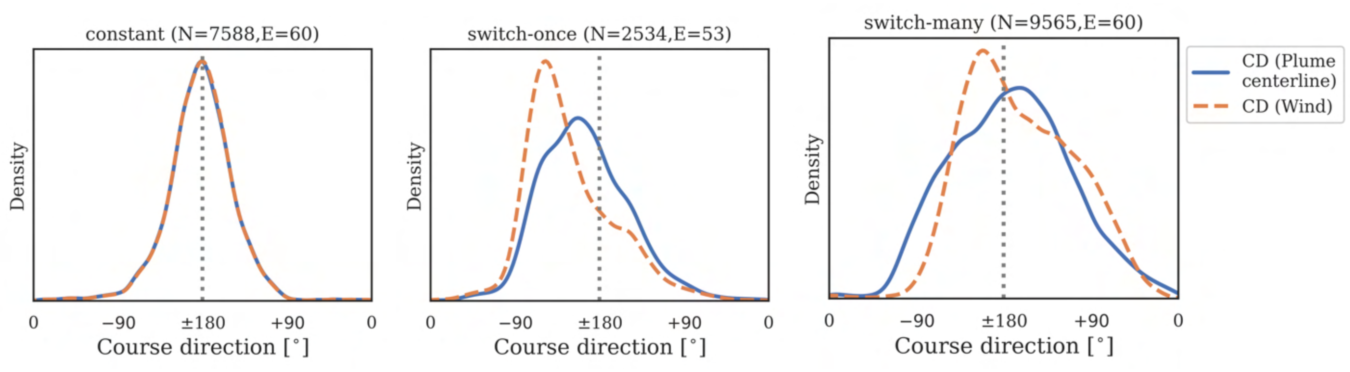

Figure SI9: Empirical course-direction (CD) distribution - Agent 2

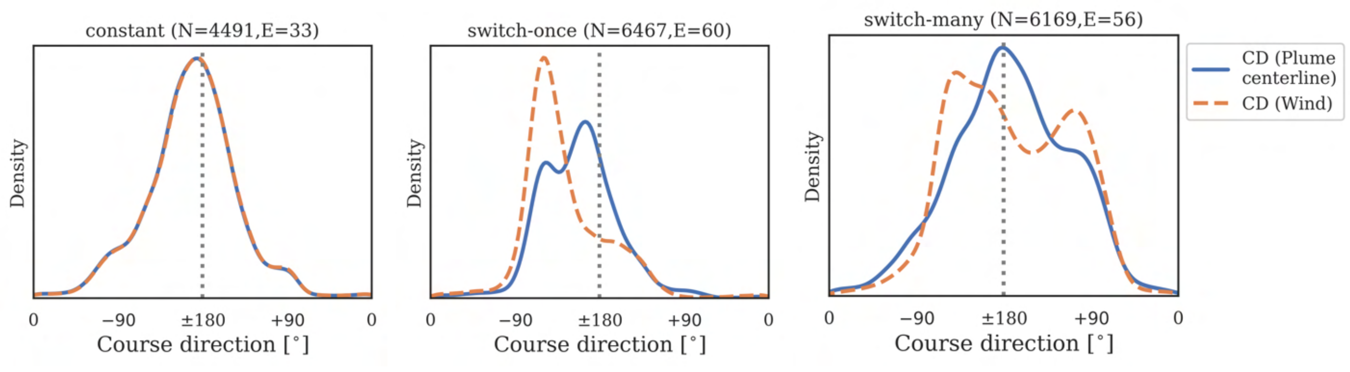

Figure SI10: Empirical course-direction (CD) distribution - Agent 3 (Same as Figure 3)

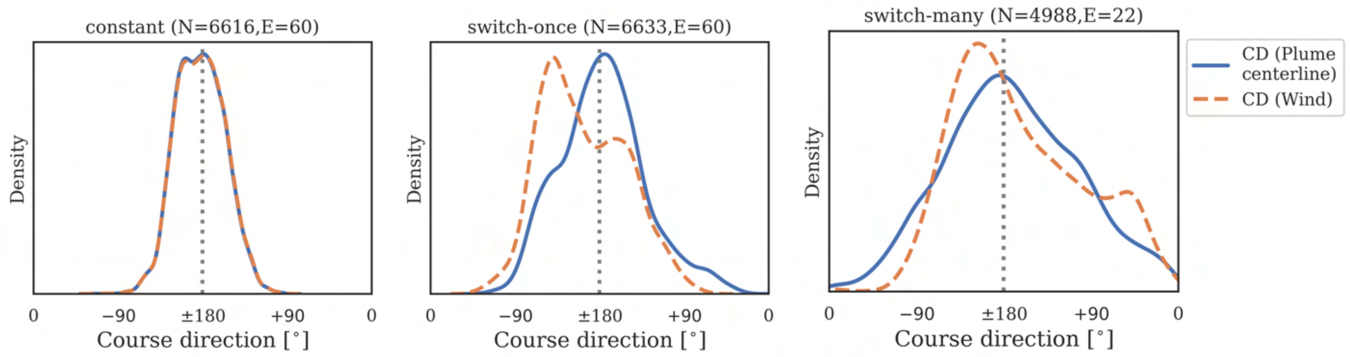

Figure SI11: Empirical course-direction (CD) distribution - Agent 4

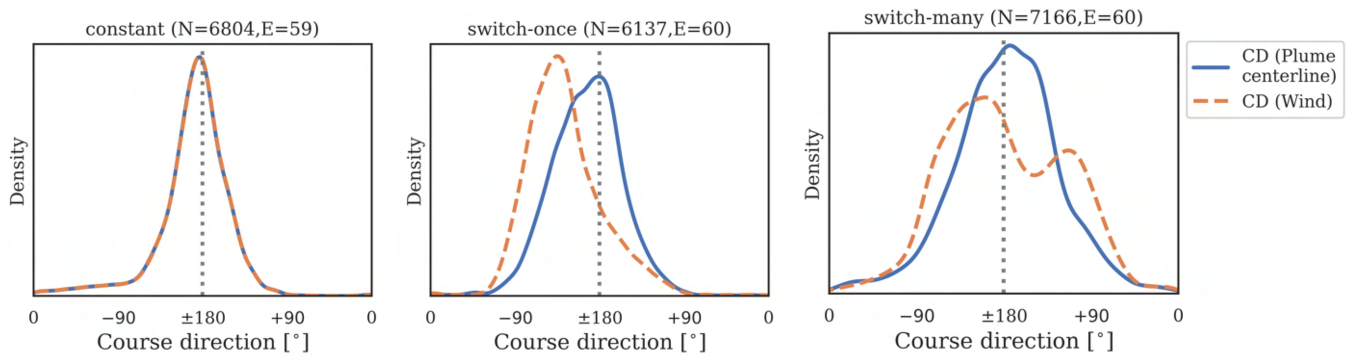

Figure SI12: Empirical course-direction (CD) distribution - Agent 5

SI4 Neural activity dimensionality and neural representations

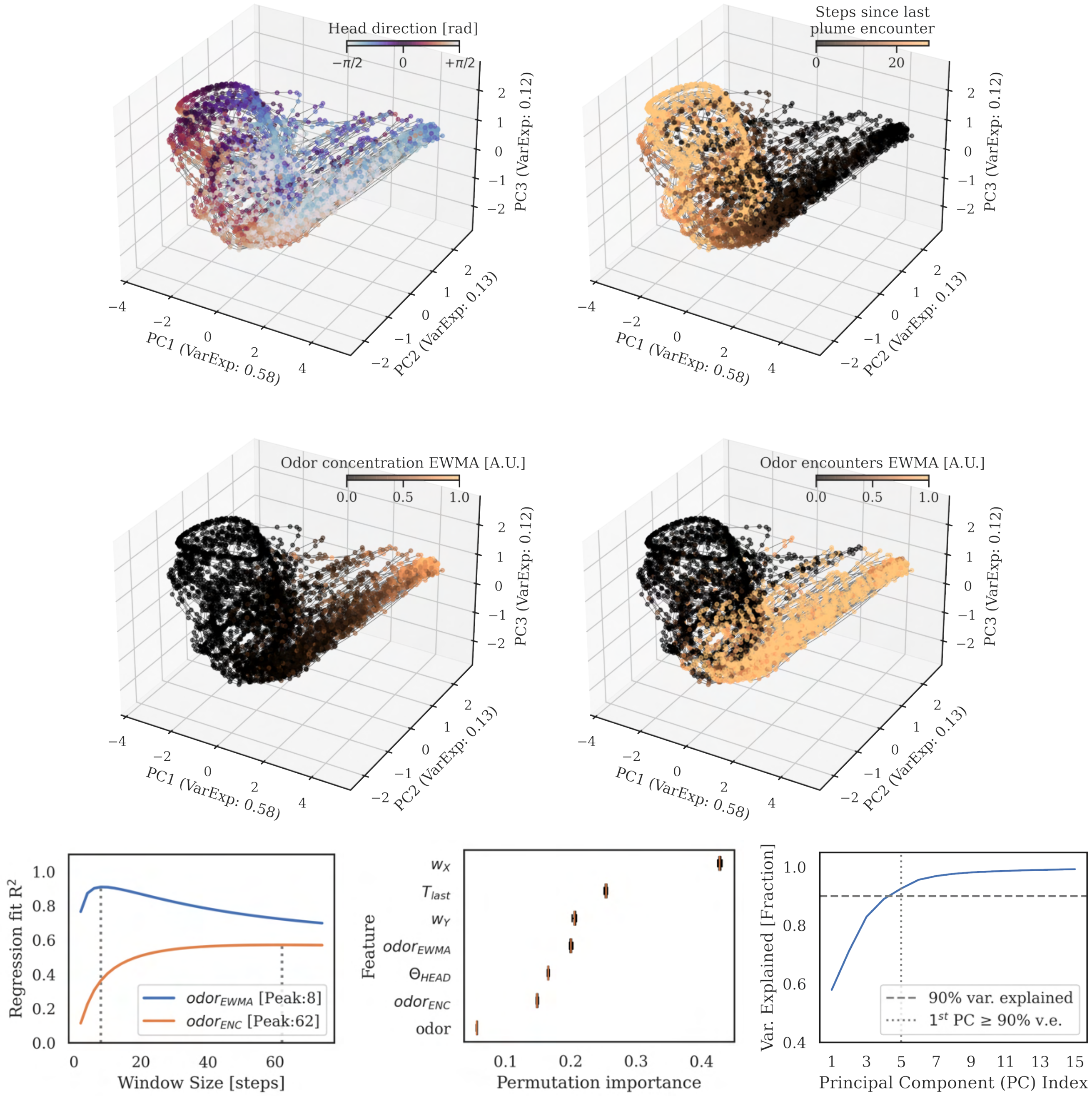

Figure SI13: Neural representations – Agent 1 (See Figure 4 for equivalent data on Agent 3 and figure details)

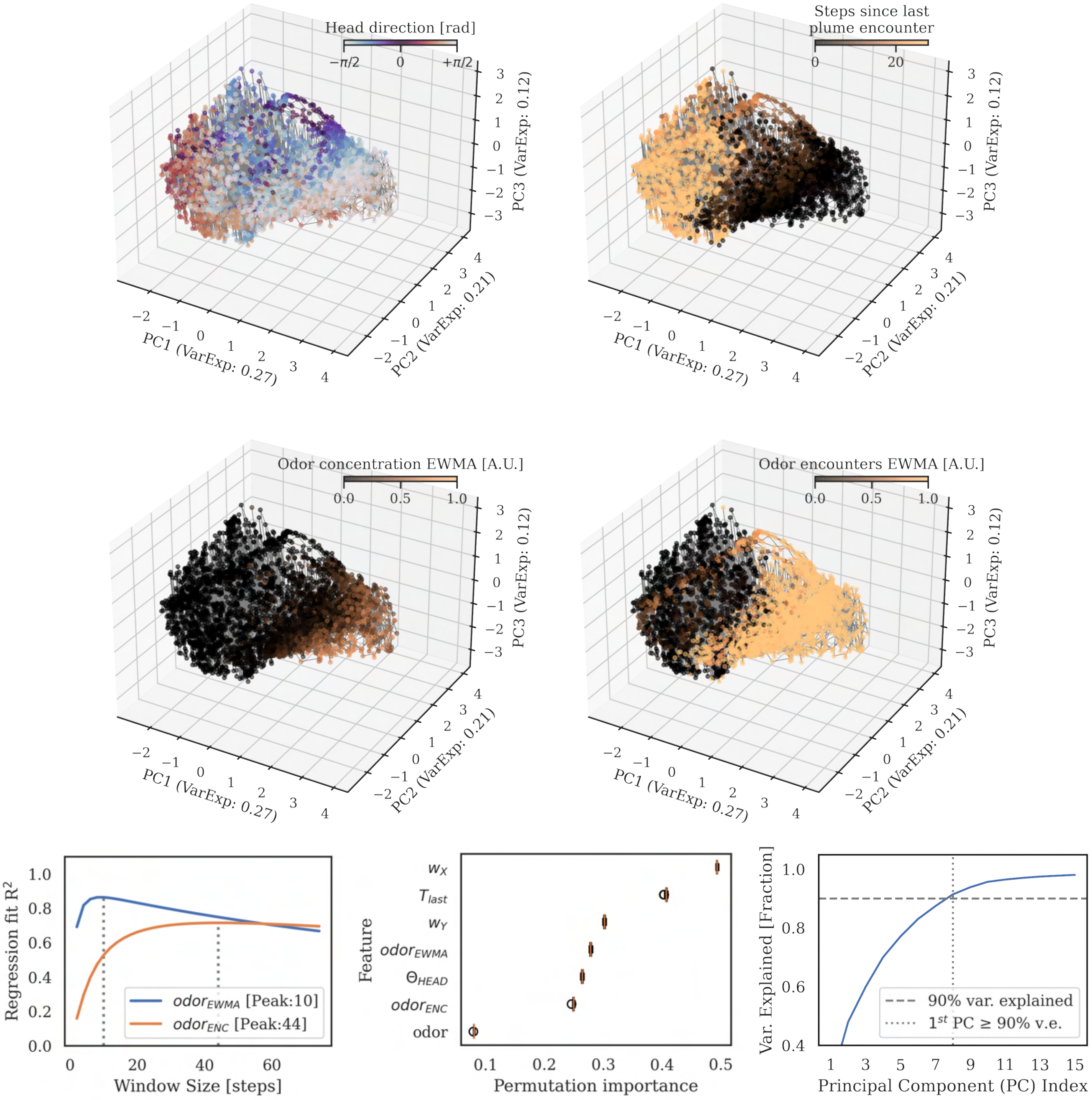

Figure SI14: Neural representations – Agent 2

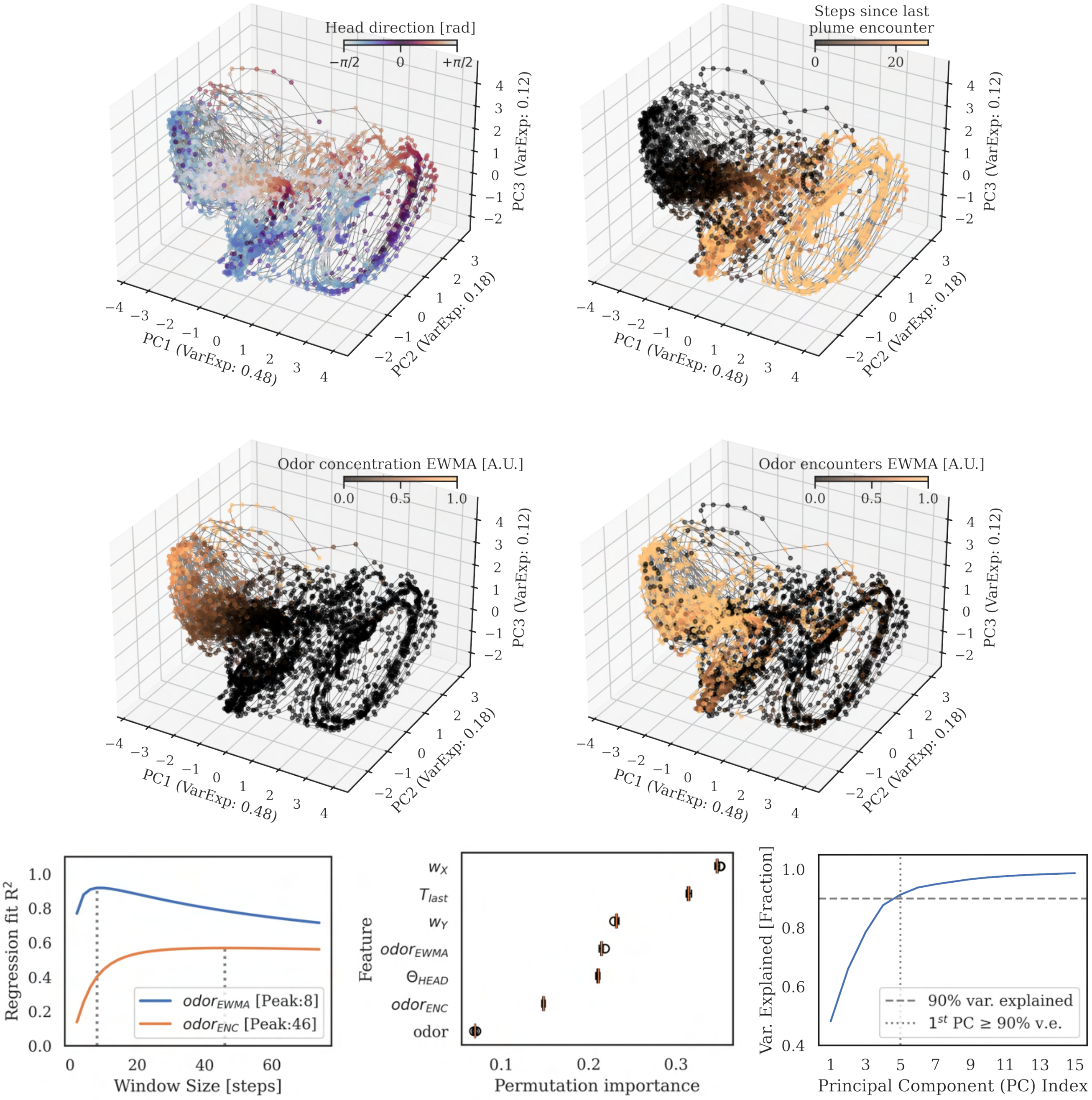

Figure SI15: Neural representations – Agent 3 (Same agent as in Figure 4)

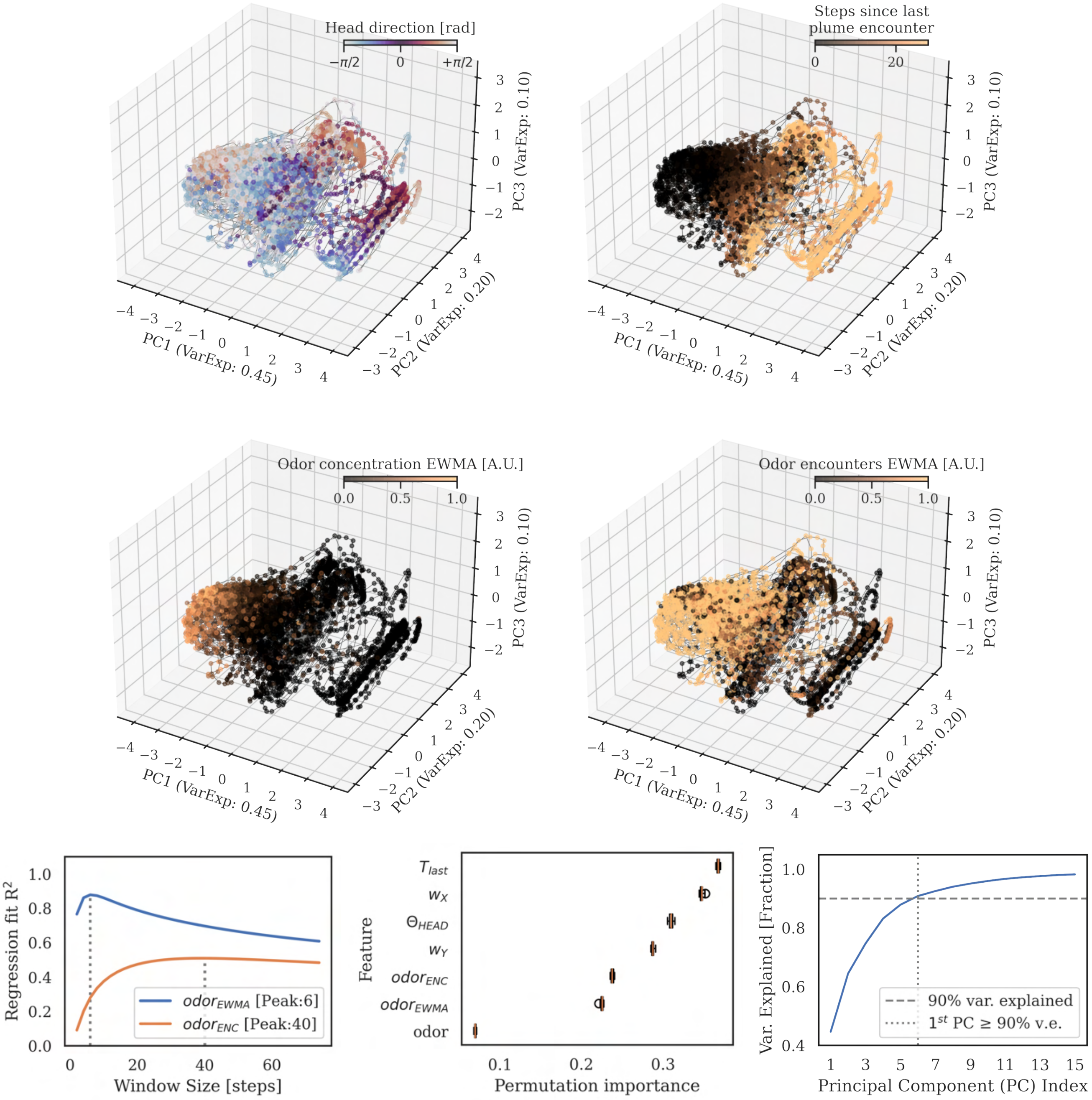

Figure SI16: Neural representations – Agent 4

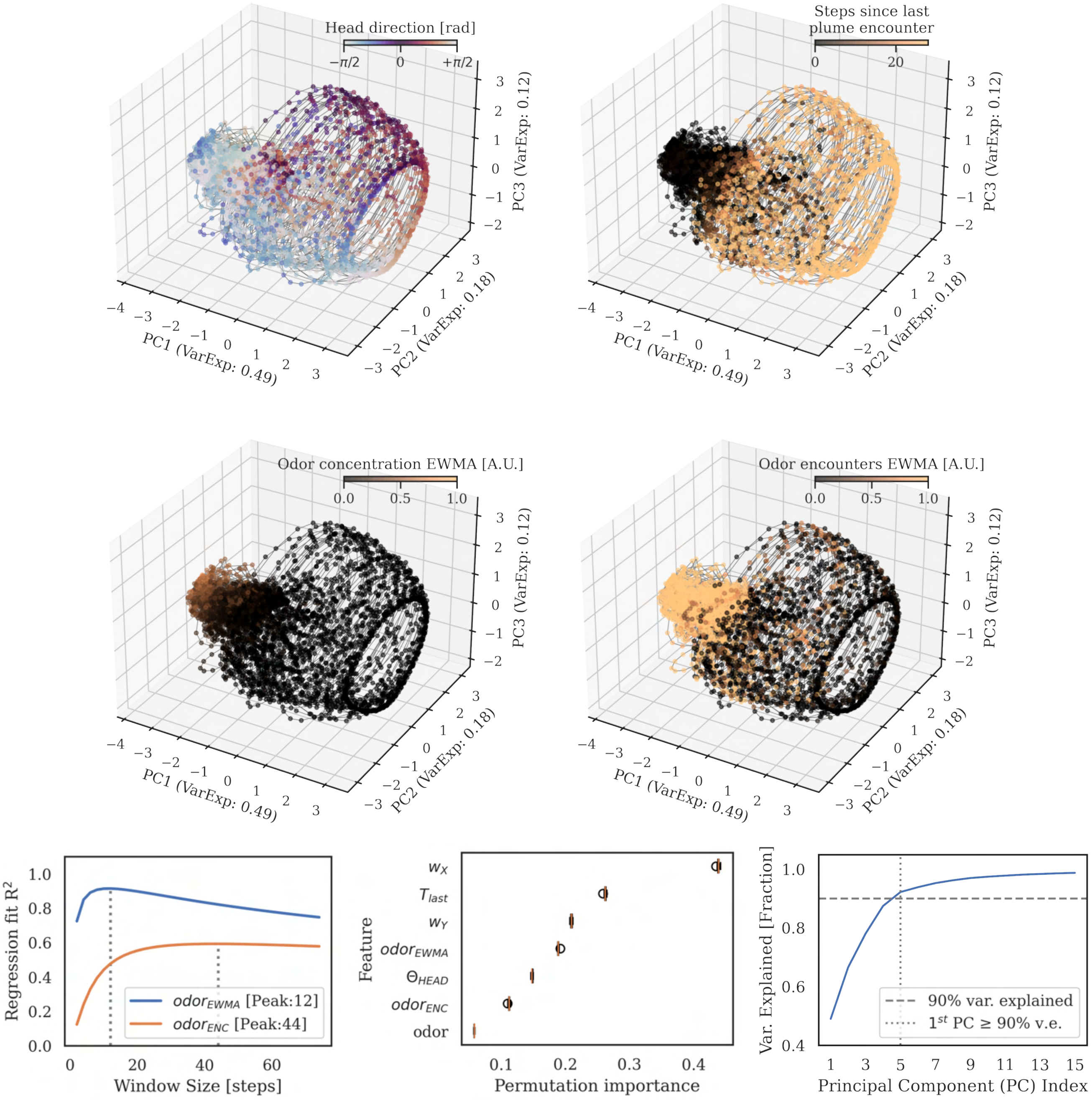

Figure SI17: Neural representations – Agent 5

SI5 Structured neural dynamics

Agent	Agent ID	Limit-cycle period
RNN 1	2760377	19 steps (0.76 s)
RNN 2	3199993	NA (clear periodic structure not observed)
RNN 3	3307e9	17 steps (0.68 s)
RNN 4	541058	28 steps (1.12 s)
RNN 5	9781ba	18 steps (0.72 s)

Table SI1: Limit cycle periods for each RNN agent

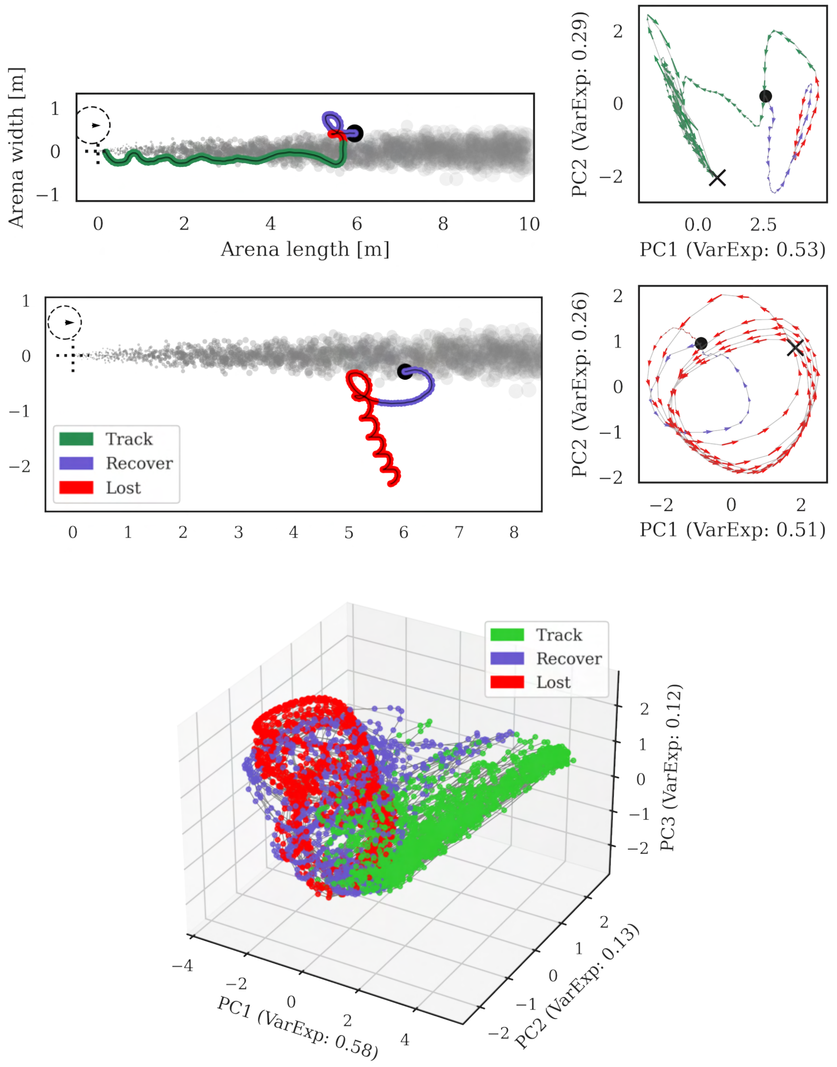

Figure SI18: Neural dynamics – Agent 1 (See Figure 5 for equivalent data on Agent 3 and figure details)

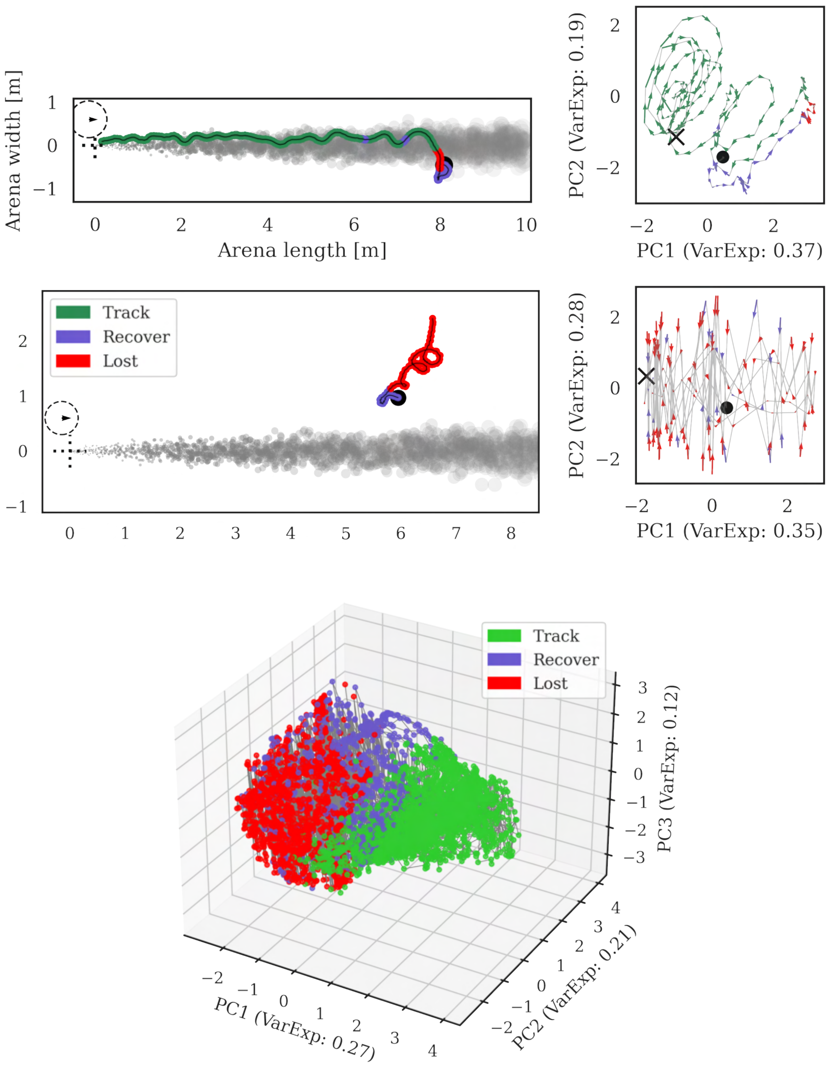

Figure SI19: Neural dynamics – Agent 2

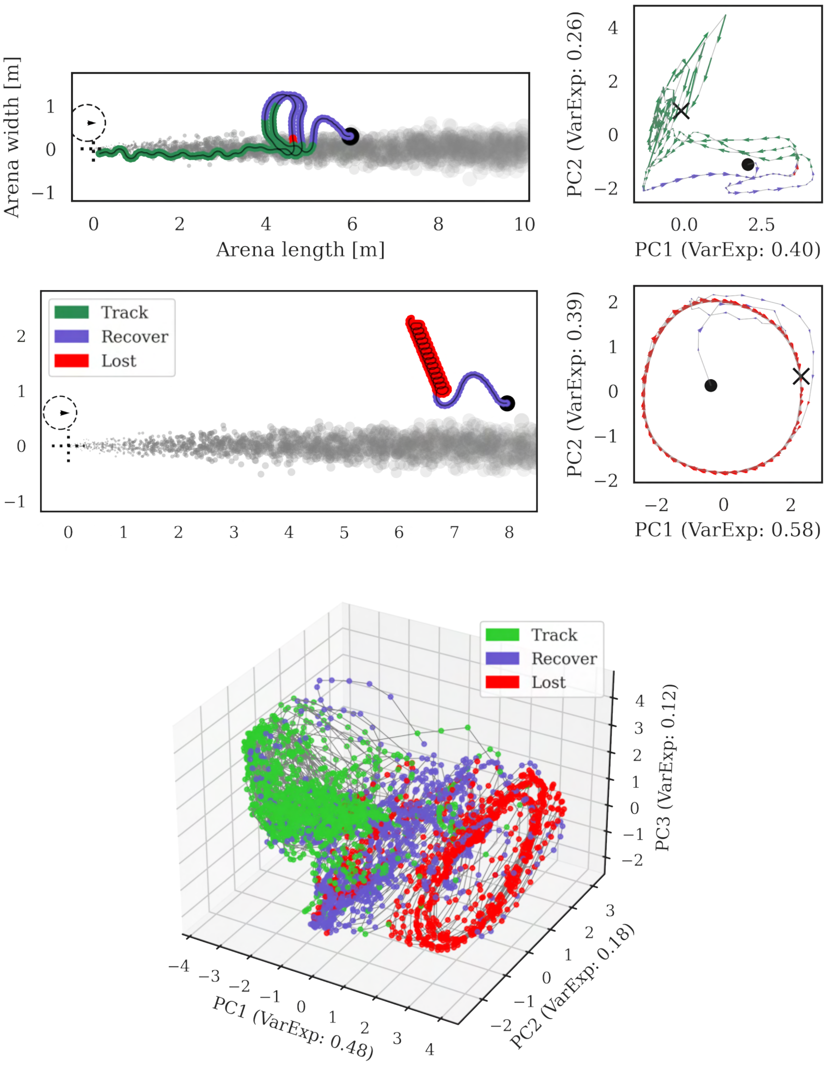

Figure SI20: Neural dynamics – Agent 3 (same as Figure 5)

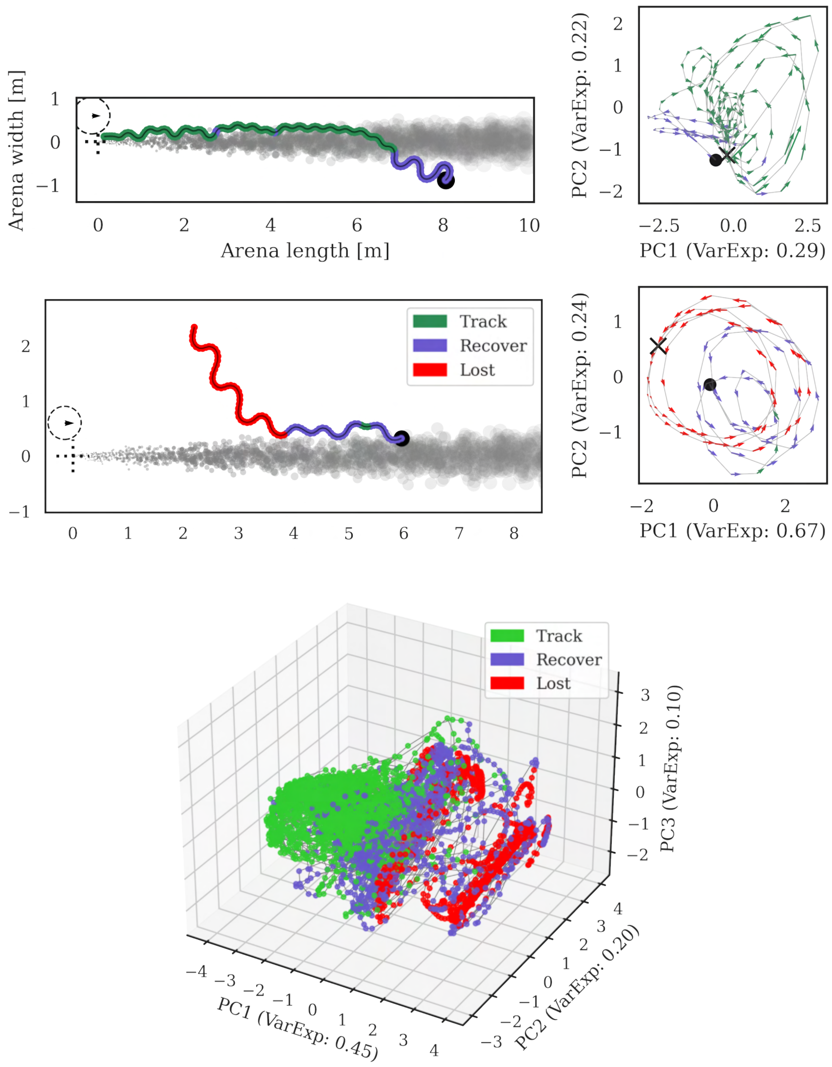

Figure SI21: Neural dynamics – Agent 4

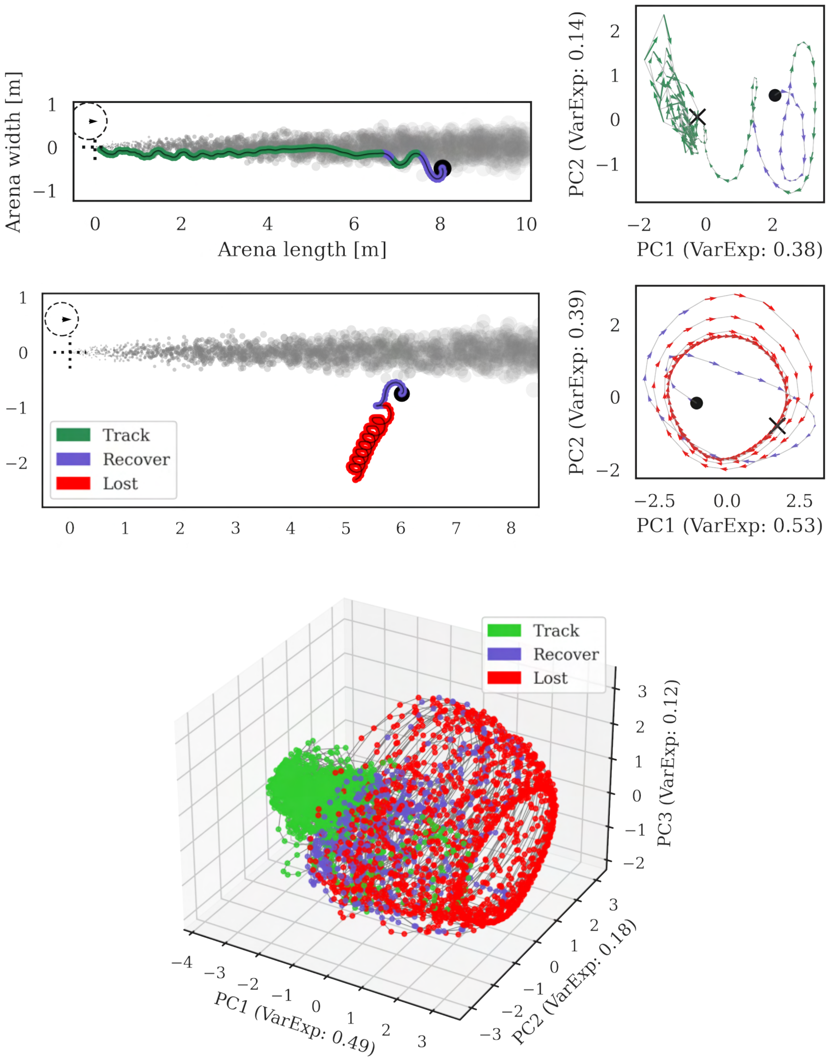

Figure SI22: Neural dynamics – Agent 5

SI6 RNN connectivity and stimulus integration timescales

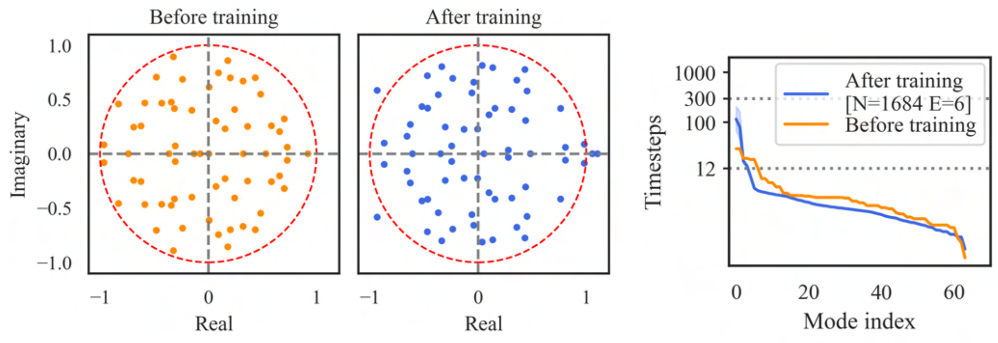

Figure SI23: Eigenspectra of $\mathbf{W}_h$ before and after training, and stimulus integration timescales – Agent 1 (compare with Agent 3 in Figure 6)

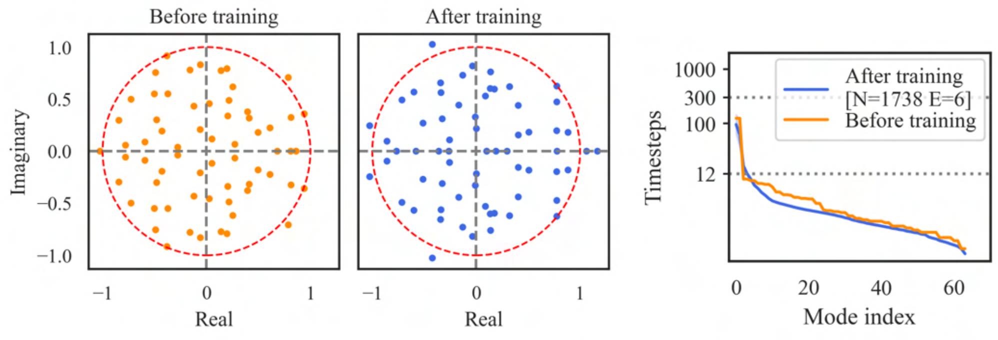

Figure SI24: Eigenspectra of $\mathbf{W}_h$ before and after training, and stimulus integration timescales – Agent 2

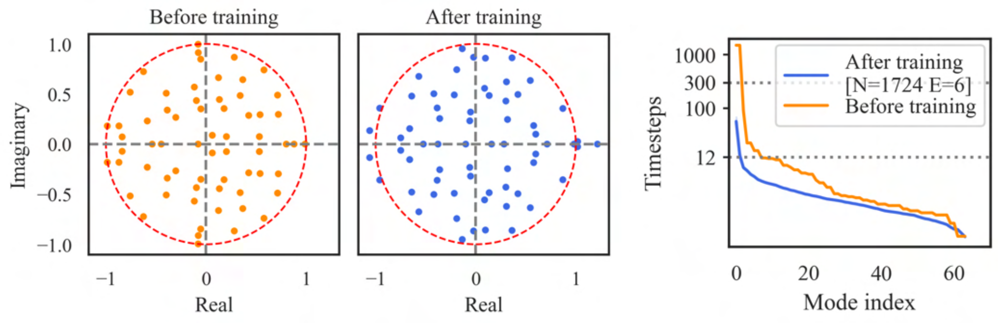

Figure SI25: Eigenspectra of $\mathbf{W}_h$ before and after training, and stimulus integration timescales – Agent 3 (same as Figure 6)

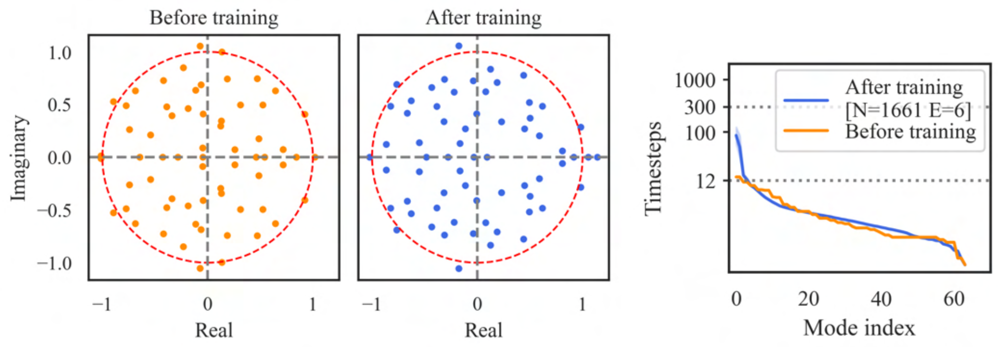

Figure SI26: Eigenspectra of $\mathbf{W}_h$ before and after training, and stimulus integration timescales – Agent 4

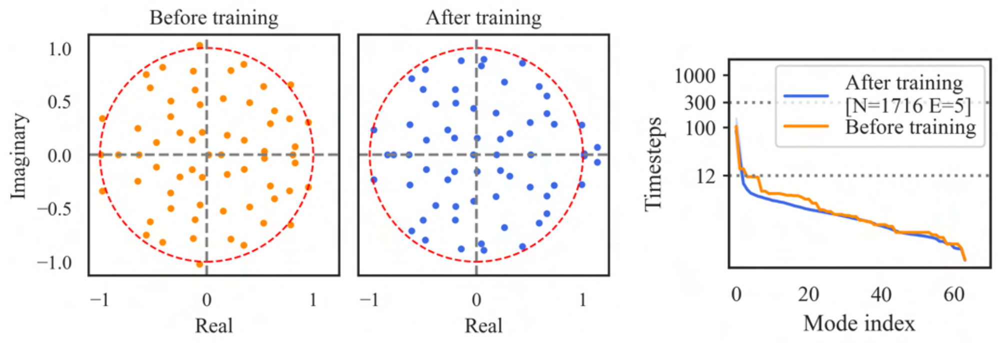

Figure SI27: Eigenspectra of $\mathbf{W}_h$ before and after training, and stimulus integration timescales – Agent 5

SI7 Additional figures for reduced (0.5x) radial diffusion-rate plumes

The four figures below are similar to Figure 2 in the main manuscript, except for that all plume simulations use a reduced (0.5x) radial diffusion-rate to encourage highly intermittent odor encounters. Trajectories have been chosen from successful episodes of RNN Agent 3 across four plume configurations. As can be seen, trained agents successfully localize the odor source with these more intermittent plumes and tracking behaviors are qualitatively no different than those seen on higher puff-density (higher radial diffusion-rate) plumes.

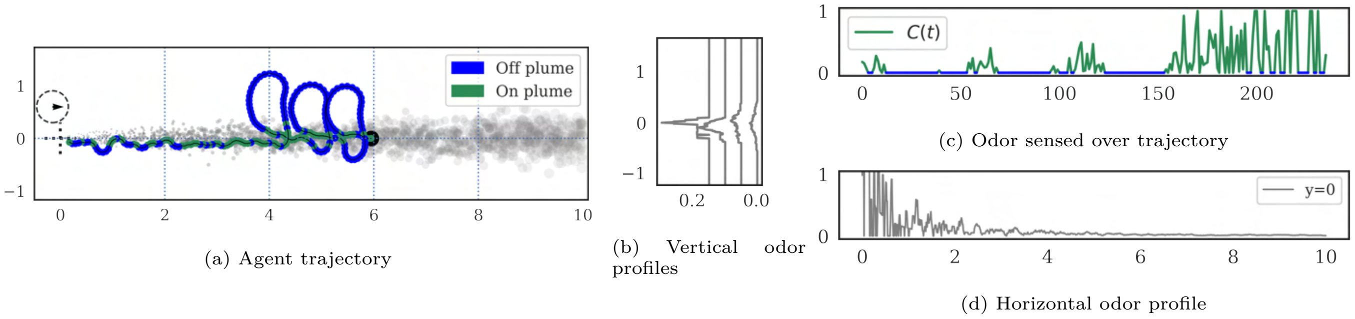

(a) Agent trajectory

(b) Vertical odor profiles

(c) Odor sensed over trajectory

(d) Horizontal odor profile

Figure SI28: Constant wind direction

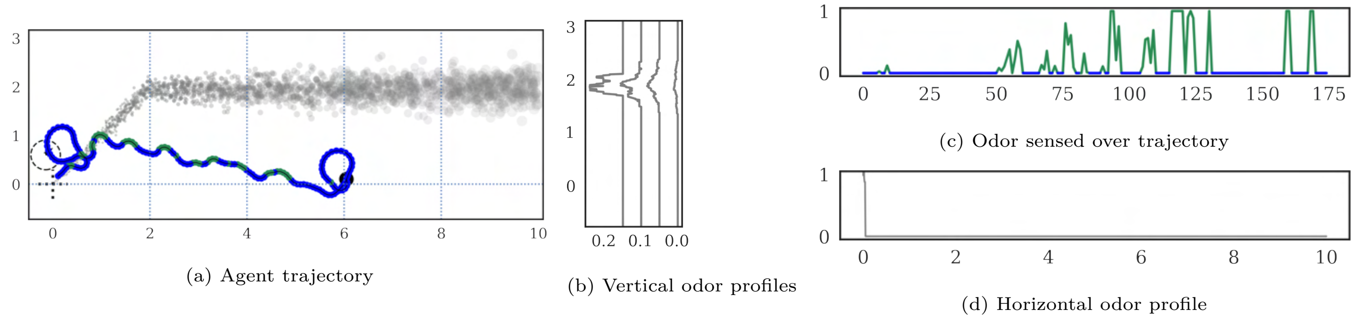

(a) Agent trajectory

(b) Vertical odor profiles

(c) Odor sensed over trajectory

(d) Horizontal odor profile

Figure SI29: Wind direction switches once

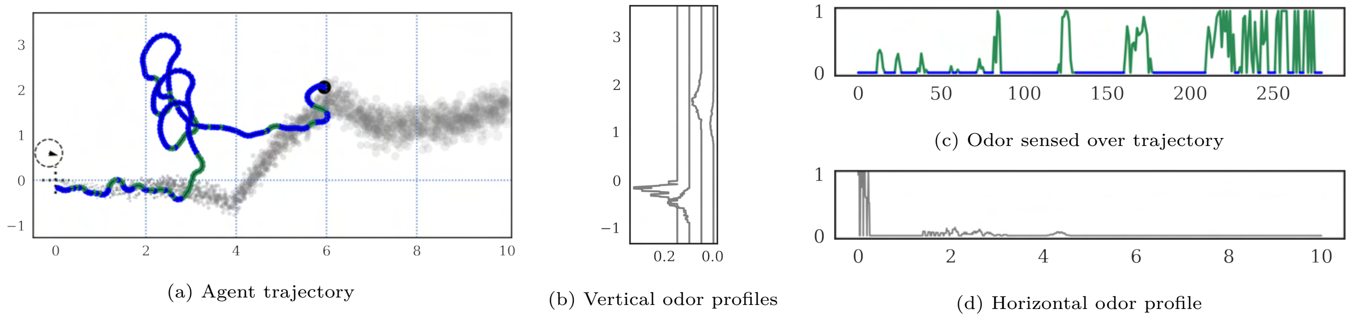

(a) Agent trajectory

(b) Vertical odor profiles

(c) Odor sensed over trajectory

(d) Horizontal odor profile

Figure SI30: Wind direction switches many times

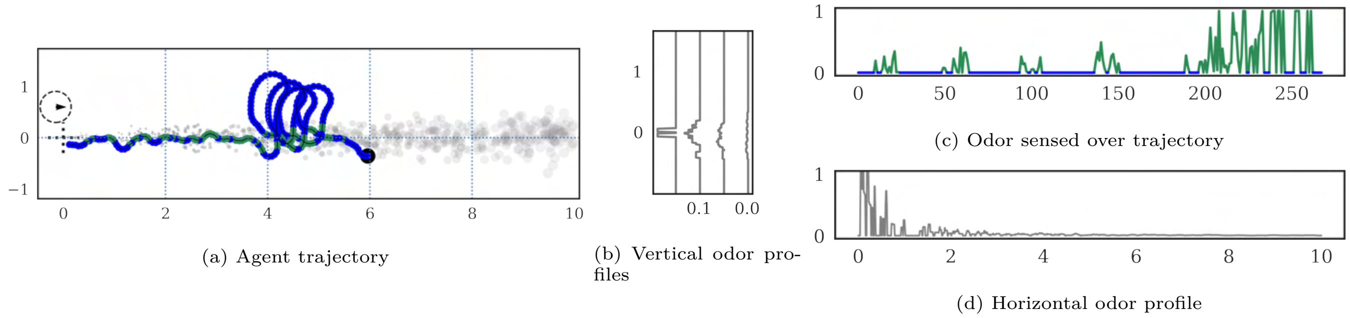

(a) Agent trajectory

(b) Vertical odor profiles

(c) Odor sensed over trajectory

(d) Horizontal odor profile

Figure SI31: Sparser plume (0.4x birthrate, 0.5x radial diffusion-rate) and constant wind direction (Same as Figure 2c)

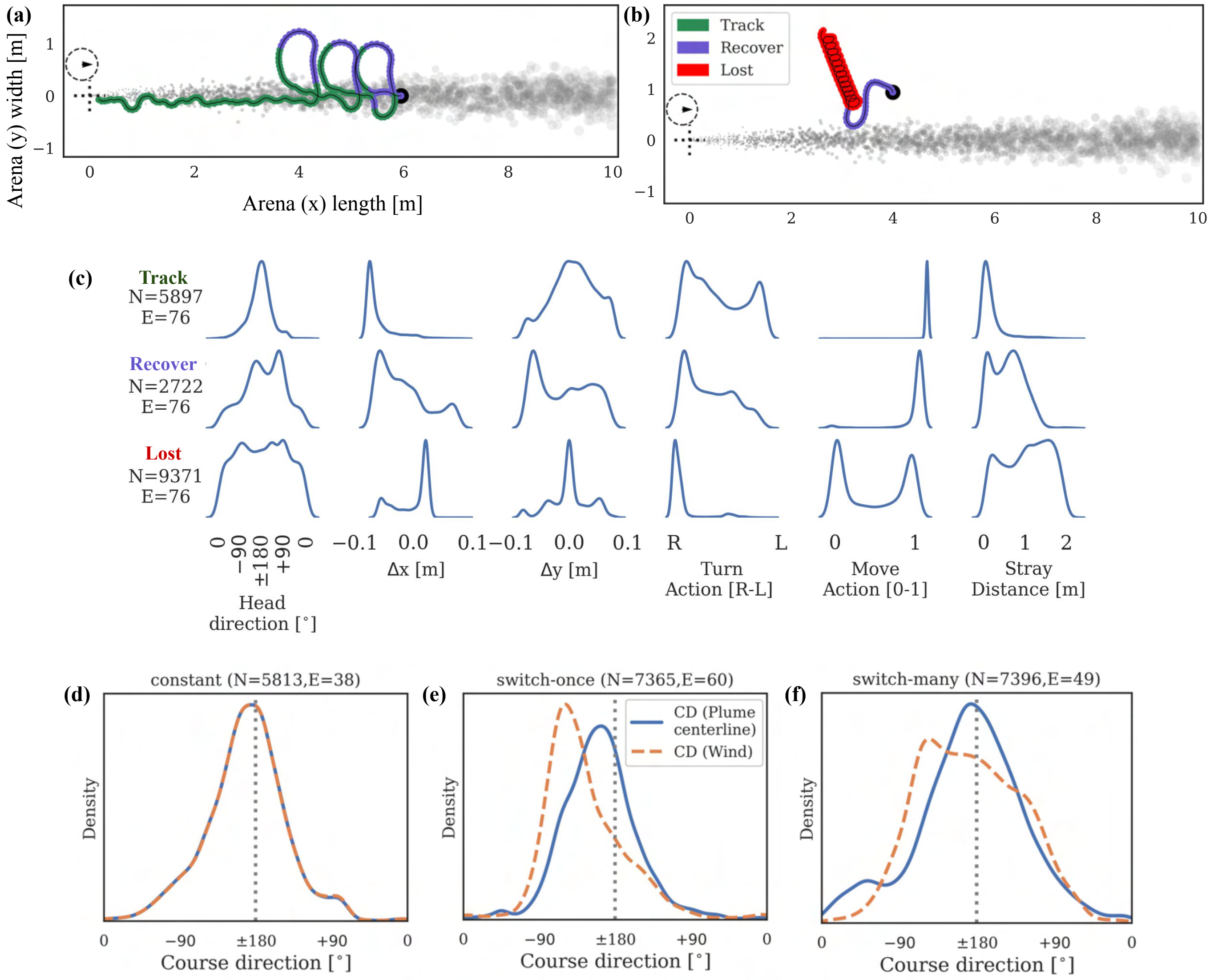

Figure SI32: Figure similar to Figure 3 in the main manuscript, except for that all plume simulations use a reduced (0.5x) radial diffusion-rate to encourage highly intermittent odor encounters. We see no qualitative differences with respect to higher puff-density (higher radial diffusion-rate) plumes. All plots are for RNN Agent 3.

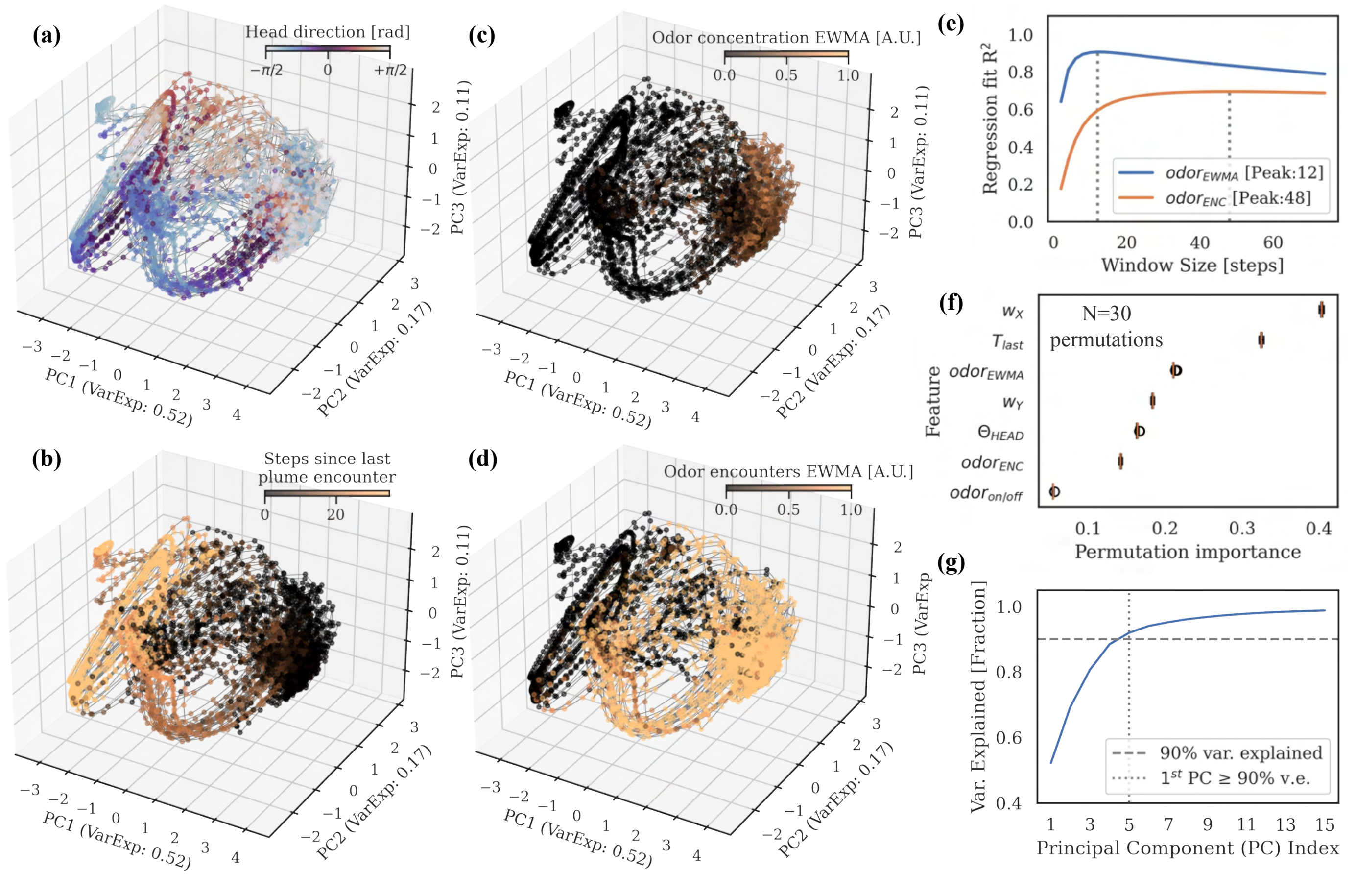

Figure SI33: Figure similar to Figure 4 in the main manuscript, except for that all plume simulations use a reduced (0.5x) radial diffusion-rate to encourage highly intermittent odor encounters. We see a qualitative difference in PCA plots of the neural dynamics, in that they now seem to highlight dynamics associated with the 'recover' regime (and to some extent, the 'lost' regime) more. We believe this is because the underlying trajectories indeed have more recover/lost regime type behaviors due to increased intermittency of odor encounters. No qualitative differences with respect to Figure 4 are seen in regards to behaviorally relevant quantities represented. All plots are for RNN Agent 3.

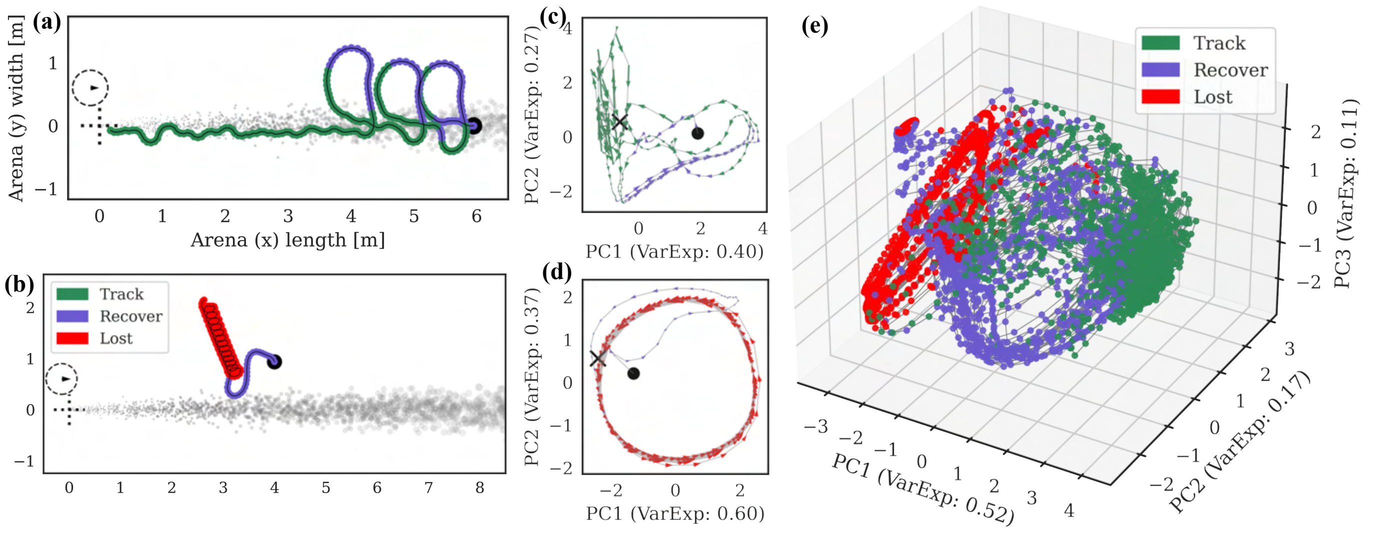

Figure SI34: Figure similar to Figure 5 in the main manuscript, except for that all plume simulations use a reduced (0.5x) radial diffusion-rate to encourage highly intermittent odor encounters. We see no qualitative differences with respect to Figure 4 in either behaviors or their representations. Neural activity PCA plot highlights dynamics associated with the 'recover' and 'lost' behaviors due to distribution shift as also seen in Figure SI33. All plots are for RNN Agent 3.

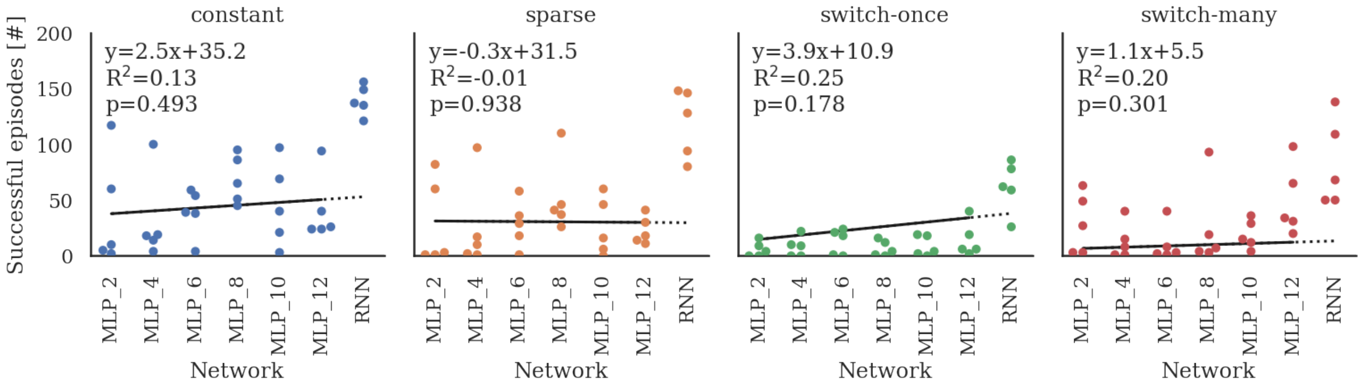

Figure SI35: Figure similar to Figure 6 in the main manuscript, except for that all plume simulations use a reduced (0.5x) radial diffusion-rate to encourage highly intermittent odor encounters. For fairness of comparison with Figure 6, we have run our behavior assay on all 14 trained agents of each architecture type (RNNs and MLPs) using reduced (0.5x) radial diffusion-rate plumes. We then re-selected the top-5 performing agents for each architecture type to generate the above figure (see Methods for behavior assay and agent selection details). We observe that 2 out of the top-5 RNNs used in Figure 6 are different from those selected here (no comparable bookkeeping was done for the MLPs). We roughly see the same trends in this plot as in Figure 6, in that RNNs clearly outperform MLPs across plume configurations. Trends across MLPs show lesser statistical significance, possibly because MLP training produced agents that are less robust to domain shift (reduced radial diffusion rates) than RNNs. Future work could explore retraining all agents across all architectures on plume with reduced (0.5x) puff radial diffusion-rates for better generalization.
